# Metabolite profiling of ascidian *Styela plicata* using LC–MS with multivariate statistical analysis and their antitumor activity

**DOI:** 10.1080/14756366.2016.1266344

**Published:** 2017-02-24

**Authors:** Satheesh Kumar Palanisamy, Daniela Trisciuoglio, Clemens Zwergel, Donatella Del Bufalo, Antonello Mai

**Affiliations:** a Department of Chemical, Biological, Pharmaceutical and Environmental Science, University of Messina, Messina, Italy;; b Department of Research, Advanced Diagnostics and Technological Innovation, Regina Elena National Cancer Institute, Rome, Italy;; c Department of Drug Chemistry and Technologies, Sapienza University, Rome, Italy;; d Pasteur Institute, Cenci Bolognetti Foundation, “Sapienza” University, Rome, Italy

**Keywords:** Ascidian, apoptosis, biomolecules, cancer, metabolomics

## Abstract

To identify the metabolite distribution in ascidian, we have applied an integrated liquid chromatography– tandem mass spectrometry (LC–MS) metabolomics approach to explore and identify patterns in chemical diversity of invasive ascidian *Styela plicata*. A total of 71 metabolites were reported among these alkaloids, fatty acids and lipids are the most dominant chemical group. Multivariate statistical analysis, principal component analysis (PCA) showed a clear separation according to chemical diversity and taxonomic groups. PCA and partial least square discriminant analysis were applied to discriminate the chemical group of *S. plicata* crude compounds and classify the compounds with unknown biological activities. In this study, we reported for the first time that a partially purified methanol extract prepared from the ascidian *S. plicata* and *Ascidia mentula* possess antitumor activity against four tumor cell lines with different tumor histotype, such as HeLa (cervical carcinoma), HT29 (colon carcinoma), MCF-7 (breast carcinoma) and M14 (melanoma). *S. plicata* fraction SP-50 showed strong inhibition of cell proliferation and induced apoptosis in HeLa and HT29 cells, thus indicating *S. plicata* fraction SP-50 a potential lead compound for anticancer therapy. The molecular mechanism of action and chemotherapeutic potential of these ascidian unknown biomolecules need further research.

## Introduction

Ascidian species possess a broad range of biodiversity of marine natural products (MNPs) because of their great ability to synthesize bioactive substances and serve as a vital source of new therapeutics. Due to their richness in biomolecules or secondary metabolite production, ascidians represent an important beginning for the discovery of new marine drugs and could be used as novel chemical scaffolds^1^. Interestingly, in Japan and Korea, ascidians are used as commercial seafood (e.g. *Styela plicata, Microcosmus exasperates, Halocynthia rortezi*) because of their high content of proteins, amino acids and micronutrients^2^^,^^3^. Hence, it is important to study their chemical diversity, pharmacological properties when considering their food applications. The marine ascidians metabolome possesses valuable resource of MNPs and another organism such as sponge, corals and algae are likely produced much more metabolites than have been discovered so far^1^.

In analytical chemistry, metabolomics is an emerging field of systematic study of the unique chemical fingerprints of flora and faunal living cells and biofluids^4^^,^^5^. Metabolomics using mass spectrometry (MS) techniques allows qualitative and quantitative profiling of small biomolecules in marine organisms. This novel approach can be applied to reveal metabolic differences between ascidian species, to find differences in their profiles and to identify novel potential chemical lead for clinical biomarker discovery. Mass spectrometry-based metabolomic profiling provides a potent approach to quantifying and identifying the chemical diversity of living organisms and developing metabolic maps. It has been wildly applied in food and nutritional science, pharmacology and toxicology research, functional genomics, drug discovery to clinical development of new drugs and quality control particularly of chemotaxonomic identification of flora and fauna^6^. Liquid chromatography–mass spectrometry (LC–MS) coupled with UV detection has traditionally lead biodiscovery efforts, but generally only detects highly abundant compounds and yields little structural information for dereplication of known compounds^7^. MS-based metabolomic platforms in MNPs research have increased the dynamic range of compound detection and dramatically decreased the quantity of material required. In spite of these advantages, the biggest challenge of MS-based metabolic profiling is processing and prioritizing the metabolomics data sets before the determination of the chemical structure of these metabolites^6^. A typical metabolomics profiling requires an enormous number of samples to generate the results that are statistically rigorous. Besides, highly sensitive and accurate instrumentation and powerful software tools (e.g. XCMS-METLIN) are essential to address the vast amount of data generated by these experiments. The recent development in the field of natural products chemistry and LC–MS/NMR-based metabolomics research on marine origin secondary metabolites exhibits diverse range of biological properties for developing new therapies to improve the health of individuals across the universe suffering from various deadly diseases such as infectious disease malaria, HIV, neurological and immunological diseases and cancer. The application of LC–MS-based metabolic profiling of biological systems has gained more extensive use in identifying drug metabolite, developing metabolite maps and lending clues mechanism of bioactivation^6^. However, the knowledge of the metabolite accumulates in different Mediterranean ascidians chemical diversity is scarce.

In this study, we investigated the metabolite distribution of invasive ascidian *S. plicata* and Mediterranean ascidian *A. mentula* using LC–MS-based metabolomics method^8^ and multivariate statistical analysis with the aim of revealing unknown biomolecules from the ascidian *S. plicata*. In an earlier study, Halouska et al.^9^ predicted the *in vivo* mechanism of action for drug leads against antitubercular activity using NMR metabolomics and orthogonal partial least square-discriminant analysis (OPLS-DA). In his study, he reported that compound separated from known drugs in the OPLS-DA scores plot, could be inferred new mechanism of action and has potentially valuable a new antibiotic agent. In this study, we have applied LC–MS metabolic profiling and partial least square-discriminant analysis (PLS-DA) to profile *in vitro* mechanism of action of known pharmacological potencies of metabolites identified in ascidian *S. plicata*. Mainly, we are processed to apply this information to classify the ascidian metabolites with known and unknown pharmacological potencies demonstrated antibacterial and antitumor action.

Recently, the European Network of Cancer Registries has reported more than 24 million cases of cancer diseases that have spread in 27 European nations. In the year 2012, around 3.45 million new cancer cases and 1.75 million deaths from cancer have been estimated in Europe^10^. Biomolecules derived from ascidian has aid potential candidate as anticancer drugs and for the development of novel therapeutic targets. For instance, the most well-known compound, ecteinascidin 743 (Yondelis^®^), isolated from the ascidian *Ecteinascidia turbinate* and marketed by PharmaMar was approved by the European Commission and Food and Drug Administration (FDA) to treat soft-tissue sarcomas and ovarian cancer disease. Another important antitumor drug plitidepsin (Aplidin^®^), isolated from *Aplidium albicans*, is in clinical usage for the treatment of acute lymphoblastic leukemia and several other ascidian MNPs are at various stages of clinical trials, biotechnological and industrial applications^11^.

The objectives of this study were to assess metabolic profiling of Mediterranean ascidian using LC–MS combined with multivariate statistical analysis (PCA, PLS-DA) according to their mass spectral variables and classify the metabolites of known and unknown pharmacological application. In addition, we aimed to identify active fractions using bioassay-guided fractionation, structure elucidates active biomolecules and screen their antitumor efficacy against cervical, colon cancer and other tumor cell lines using MTT bioassay to find the antitumor activity of ascidian species.

## Materials and methods

### Chemicals

MeOH – methanol, CHCl_3_ – chloroform, CH_2_Cl_2_ – dichloromethane, Na_2_SO_4_ – sodium sulfate, formic acid, TFA – trifluroacetic acid (Sigma-Aldrich, St. Louis, MO) were used as solvents for the extraction of crude extracts preparation and successive partition of the aqueous phase. DMSO – dimethyl sulfoxide, isopropanol, PBS – phosphate-buffered saline and PI – propidium iodide were used for screening biological activity of ascidians.

### Sample collection and extraction

Ascidians were collected by snorkeling in Lake Faro, Messina coast, Italy at a depth 3–5 m and identified to the lowest taxonomic level^12^. A voucher specimen is deposited at the Department of Biological and Environmental Science, Messina, Italy. Crude extracts from the nonindigenous ascidian *S. plicata* (weight 42.3 g) and Mediterranean-native species *A. mentula* (24 g) have been extracted by Soxhlet extraction and ultra-sonication method using MeOH/CHCl_3._ The chemical characterization of both crude compounds was determined using Fourier transform infra-red spectroscopy (FTIR) and ^1^H-NMR techniques. The IR spectral data were examined by^13^, to differentiate the both ascidian based on the functional group of chemical components (Table S1 is given in Supplementary Information). However, nontargeted FTIR fails to monitor the species-specific metabolites and variation of chemical structure because of the limited specificity and sensitivity.

### LC–MS-based metabolomics

LC–MS spectrum analysis of ascidian crude extracts (CE) was performed on a Waters ZQ-LC–MS electrospray ionization (ESI) single quadrupole mass spectrometer using a C_18_ column 3.3 μm, 15 cm ×2.1 mm with following mobile phases: (A) H_2_O 0.1% formic acid and (B) MeOH 0.1% formic acid (sample input and acquisition; 0.1 ml/min flow rate and 5–20 μl injection volume). For our integrated metabolite profiling of ascidian species, we utilized LC–MS mass data acquisition and an open source platform MS software XCMS-METLIN^14^. The raw data file was analyzed using XCMS software combined with METLIN, and for MS data, the following libraries were used HMDB, KEGG and LMID^14^^,^^15^. LC–MS spectral data of each single sample were collected, the data will be preprocessed before the principal component analysis (PCA). The data normalization was identified using descriptive analysis followed by log transformation and Pareto scaling method. Partial least square discriminant analysis (PLS-DA) was performed to explore and identify patterns in chemical diversity of ascidian *S. plicata*. All the statistical analyses were performed using STATISTICA (ver. 10.0; Tulsa, OK) and PAST software (ver. 2.17C; Norway) for Windows. 

### HPLC separation of fractions and compound identification

A deep brownish gum (2.5 g) obtained from *S. plicata* was dissolved in 2 ml of DMSO and the extracts were equally divided. All solvents used for high-performance liquid chromatography (HPLC) and MS were HPLC grade (Sigma-Aldrich), and the H_2_O was Millipore Milli-Q PF filtered. The fractions were further diluted with 5 ml of MeOH, 1 ml of extract was transferred onto cotton and kept 30 min to dry and independently subjected to HPLC separation on a betasil C_18_ column (5 μm, 150 × 21.2 mm). Two of these columns were connected in HPLC and the combined columns eluted with a linear gradient of H_2_O containing 1% TFA at 9 ml/min flow rate over 60 min^16^. The fractions were collected by autosampler and further acquired ^1^H-NMR of all the 60 fractions (Figure S1); combined all the fractions with a similar chemical shift. From these, the following fractions SP-8, SP-28, SP-50, SP-53, SP-55 was selected and further purified by HPLC (Figure S2) and screened against four tumor cell lines. The pyrimidine nucleoside (**1**) and long-chain fatty acid (**2**) were isolated from SP-8, SP-53 and their tentative structure was elucidated using 2D-NMR spectroscopy. ^1^H-NMR data of ascidian CE were recorded by Agilent 500 MHz NMR using 5-mm NMR tube and ^1^H-NMR, ^13^C and other 2D-NMR spectroscopy of individual fractions were acquired by Agilent 600 MHz NMR using 3-mm tube and the data were analyzed by MestreNova software, version 10.01 (Mestrelab Research).

### Antitumor activity of ascidian compounds

#### Cell culture

*BJ*-EHLT (human fibroblasts), HeLa (cervical carcinoma), MCF7 (breast carcinoma) cells were cultured in Dulbecco's modified Eagle medium (DMEM), while HT29 (colon carcinoma), M14 (melanoma), HT1080 (fibrosarcoma) cells were cultured in Roswell Park Memorial Institute medium (RPMI medium). Cells were maintained at 37 °C, 5% CO_2_, 95% air and 100% humidity. 20 mM stock solution was prepared from crude and purified fractions dissolved in DMSO and stored at −20 °C. 1000–3000 cells in 200 μl were seeded in 96-well plates. Cells were treated for 48–72 h with concentrations ranging from 1 to 50 μM.

#### Cell viability

The effect of ascidian compounds on tumor cell proliferation was analyzed by measuring 3-(4,5-dimethyl-2-thiazolyl)-2,5-diphenyl-2*H* tetrazolium bromide dye absorbance of cells following manufacturer’s protocol. Cell viability in treated versus untreated control cells was calculated for each concentration of drugs used as “optical density (OD) of treated cells/OD of control cells” ×100. Data were analyzed by the median-effect method to determine the concentration that causes 50% of cell viability inhibition (IC_50_). Significant differences between the mean of control versus various doses were tested by one-way analysis of variance (ANOVA), followed by Dunnett’s *post hoc* test for multiple comparisons using GraphPad Prism 6, Windows 10, CA. Statistically, significant differences were assumed at *p*< 0.05 (*p< 0.05, **p< 0.01).

#### Cell cycle and apoptosis analysis

Cell-cycle distribution by PI staining was performed as previously described^17^. Briefly, 50,000 cells were cultured in 6-well plates, treated for 72 h with concentrations ranging from 1 to 50 μM, collected by centrifugation, fixed in cold 70% ethanol and stained with a PBS solution containing PI (62.5 mg/ml; Sigma-Aldrich) and RNase A (1.125 mg/ml; Sigma-Aldrich). Samples were acquired with C6 Accuri Flow cytometry (BD Bioscience) and the percentage of cells in the different phases of cell cycle and in the sub-G1 compartment was calculated. Apoptosis was quantified by cytofluorimetric analysis staining cells simultaneously with FITC-annexin V and the nonvital dye PI (Immunological Sciences, Rome, Italy), following the manufacturer’s instruction. At the end of incubation with the respective reagents, samples were analyzed with C6 Accuri Flow cytometry (BD Bioscience). About 20,000 events were acquired and gated using forward scatter and side scatter to exclude cell debris. Bivariate analysis allows the discrimination of viable cells (FITC−/PI−), early apoptotic (FITC+), and late apoptotic or necrotic cells (FITC+/PI+).

## Results and discussion

### Comparative analysis of ascidian metabolome

The challenge of untargeted LC–MS-based metabolic profiling is to find a technological approach that allows the reproducible identification, combined with adequate statistical analysis and quantification of the highest possible number of metabolites. LC–MS metabolomic analysis was performed for the rapid identification of novel biomolecules from both ascidians *S. plicata* and *A. mentula* MeOH crude compounds. The analysis of the LC–MS chromatogram of ascidian samples with XCMS-METLIN software revealed a total of 71 metabolite peaks with containing between 105 and 1365 *m/z* in *S. plicata* and 21 metabolites peaks containing between 117 and 519 *m/z* in *A. mentula* extracts, the complete characterization of all detected metabolites would be impossible. In this study, the maximum numbers of metabolites were produced by nonindigenous ascidian *S. plicata* compared to Mediterranean-native species *A. mentula*. Fatty acids, cembranoid, pyrimidine and alkaloid compounds are the most dominant compounds in *S. plicata*. In *A. mentula,* alkanes and flavonoids are prepotent. Significant metabolic differences were observed between both ascidian species. XCMS analysis provided statistical information about the differences between two ascidian species, including *p* values, fold change and LC retention time comparisons. Features considered to be present in the samples met the following criteria: *p* > 0.01, low fold change (<1.5) and <0.3 min deviation in LC retention time. We defined a unique feature as one with a *p* values of 0.01 and fold change of 1.5 or higher. Mass spectrum of all the metabolite peaks was analyzed by manually to confirm the comparative analysis of XCMS study. As suggested by Sidebottom et al.^18^, we eliminated those features not meeting the specifications due to their higher possibility that could be false positives from the peak picking algorithm^15^^,^^18^. Besides, the ionization state and the presence of adducts (e.g. [M + Na]^2+^;[M + H + Na]^2+^) was evaluated to verify that the correct parent ion species was determined^18^. Relative peak metabolites identified in both ascidian species were given in Supplementary Tables S2 and S3.

From the identified metabolites, antibacterial and antiprotozoal macrolides, acetylapramycin (*m/z* 291.65) and antimalarial kabiramide B (*m/z* 483.4) were previously reported in sponge *Pachastrissa nux* collected in Thailand^19^. Another neuroactive benzomorphan small molecule, pentazocine glucuronide (*m/z* 253.61) is a kappa opioid receptor agonist was reported in *S. plicata*^20^. Another, antibiotic metabolite β-lactam dicloxacillin sodium^21^ was reported in MeOH extracts of *A. mentula*. However, this is the first report for the presence of these compounds in tunicates. Similar to this study using XCMS platform, 150 metabolite features were identified from actinomycete *Streptomyces coelicolor* M145 and discovered 16 siderophores not previously reported in this strain^18^. Besides fatty acids and polyunsaturated aldehydes are the main metabolites in the interaction of the marine organism with their environment^22^^,^^23^. In aquatic ecosystem, a highly unsaturated fatty acids are essential to transfer carbon between primary producers and consumers^24^. More abundant of fatty acid metabolites present in *S. plicata* may help to compete with other native species in the Mediterranean Sea. Interestingly, the presence of metabolite decarbamoylgonyautoxin 1 (*m/z* 138.35) and lyngbyatoxin (*m/z* 216.65) molecule were first reported in *S. plicata* which is causing paralytic shellfish poisoning (PSP)^25^ and could be utilized to biotoxin monitoring for shellfish harvesting in Lake Faro, which is completely absent in *A. mentula*. The first report of PSP syndrome in human after consumption of ascidian *Microcosmus vulgaris* was reported in Croatia^25^. Results present in this study suggest that ascidian *S. plicata* collected in Messina coast is not recommended for human consumption and could be cause serious economic loss of shellfish aquaculture. However, further studies of these biotoxins in ascidian species and continuous environmental monitoring is urgently required.

### Chemotaxonomic classification

**Figure 1.**
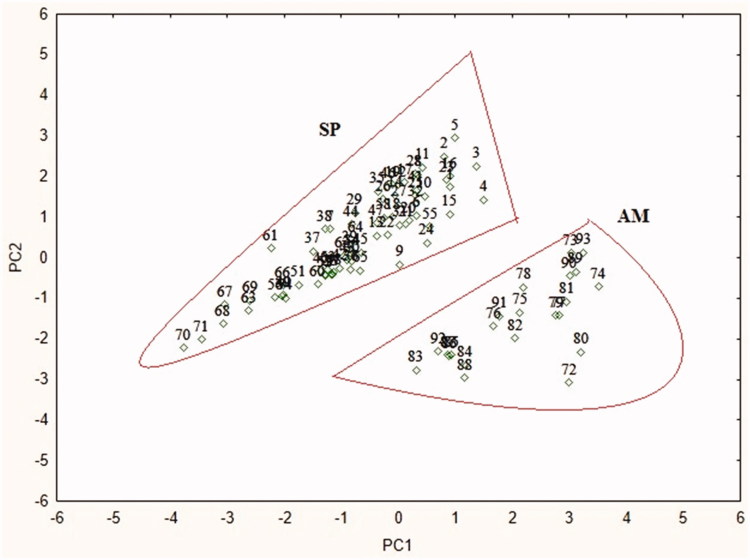
Chemotaxonomic classification of ascidian using mass spectral variables. Species SP: *Styela plicata*; AM: *Ascidia mentula.*

A total of 92 metabolites mass spectral variables, chemical group composition and taxonomic group of ascidian species were subjected to PCA. A data matrix was constructed using the taxonomic group and chemical composition as given in Tables S2 and S3. In PCA, the loading scatter plot clearly discriminates the two ascidian species separately based on the metabolites highly contribute to discriminate the samples (Figure S3). In Figure 1, the discrimination of two ascidian species can be observed clearly, the native ascidian species *A. mentula* distinctly discriminated from the invasive ascidian *S. plicata* and plotted separately. The variables of fatty acids, carotenoids were highly contributed to clear discrimination of both ascidian species. Similar results of this present study, the chemotaxonomy of marine plants and organism have been reported using GC-MS, NMR based metabolomics approach using multivariate statistical analysis^6^^,^^15^^,^^26^. Based on the results of this study, it can be concluded that PCA analysis of LC–MS mass spectral variables could be utilized as a reliable tool for species discrimination and taxonomic classification of marine ascidians.

### Multivariate statistical analysis

**Figure 2.**
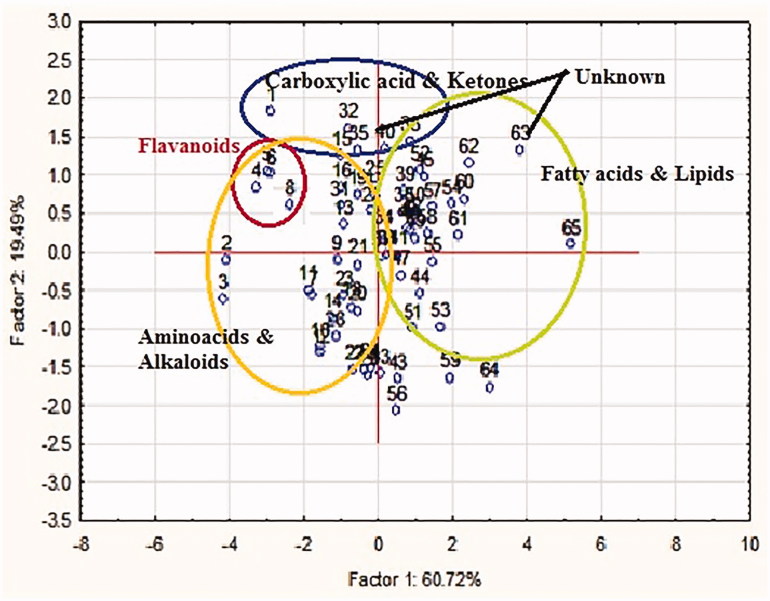
PCA demonstrating the clustering of chemical groups of known metabolites.

One of the prospective application of our untargeted metabolomic analysis in marine ascidian aims to identify potential species with novel biomolecules and classify compounds with an unknown mechanism of actions. We have chosen ascidian *S. plicata* on the basis of maximum chemical diversity and pharmacological potentials to classify their chemical compounds using multivariate statistical analysis PCA and PLS-DA^9^^,^^15^. Using relative quantification and PCA clustering of all annotated metabolites in positive ionization mode, clear differences were noted in chemical compounds of *S*. *plicata*. PCA scores plot demonstrated the clustering pattern of seven different classes of known metabolites (Figure 2). Loadings of group 1 (principal component (60.72%) and group 2 (19.49%) is complex as samples overlapped. In this study PC1, corresponding to the higher eigen value, is mostly correlated with the variable retention time, peak maximum intention and adduct molecular weight (a strong positive correlation that these axes are highly related to differences between the groups). In the first discrimination function, fatty acids and lipids discriminate compared to other metabolites reported in ascidian *S. plicata*. Prodigious, fatty acids and lipids metabolites were found more abundant in this species (Table S2). In second group PC2, amino acids, flavonoids and carboxylic acid showed very different distribution pattern in the *S. plicata*. Marshall et al.^26^ have reported metabolites, fatty acids and steroids are highly contributed to the discrimination of algae *Chattonella* sp. Similar to this present study, the metabolites of Egyptian soft corals were identified using MS/NMR-based metabolomics approach and coral metabolites were discriminated using OPLS-DA method^27^.

**Figure 3.**
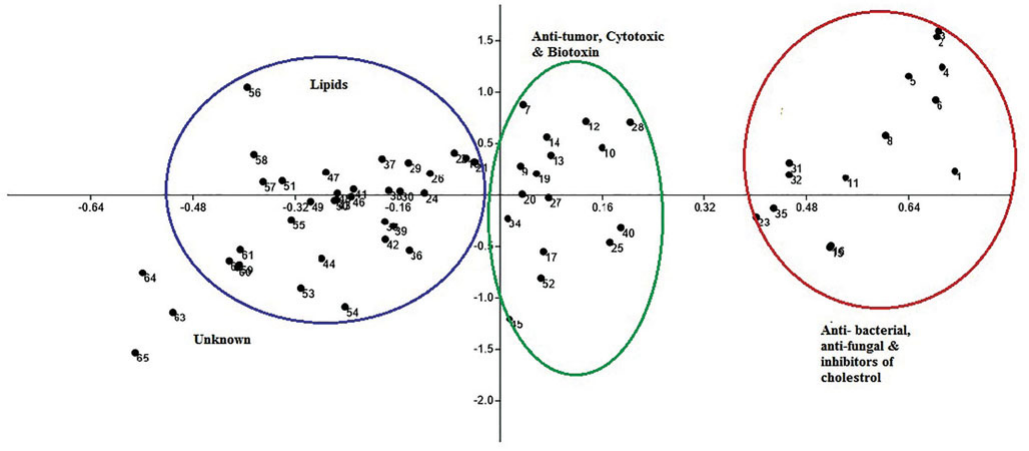
PLS-DA scores plot demonstrating the clustering pattern of *Styela plicata* metabolites with known biological potencies.

In this study, we utilized LC–MS *m/z* features of *S. plicata* and PLS-DA to profile biological potentials of known metabolites used as antibacterial, antifungal and antitumor. Herein, we aim to use this information to classify the metabolites with unknown pharmacological potentials and mechanism of action. A representative of PLS-DA scores plot demonstrates clustering pattern of chemical groups with known mechanisms and *in vitro* biological potencies of *S. plicata* (Figure 3). Each point in PLS-DA scores plot represents electron ionization of LC–MS spectrum of specific metabolites. A quality of assessment score (*R*^2^) of 0.545 and (*Q*^2^) of 0.48 was obtained, indicating an excellent and highly reliable model^9^^,^^27^. Correspondingly; PLS-DA is preferred as long as cross validation verifies a reliable model. The PLS-DA scores plot consists of three separate clustering patterns, which revealed that each group has considerably different potency on the metabolome of *S. plicata.* Importantly, fatty acids and lipids group metabolites were distinct and separate clusters from other biologically active metabolites inhibiting antibacterial, antifungal and antitumor growth (biological activity of these metabolites were retrieved from databases HMDB, KEGG, LIMD). The biomolecules with similar biological activity or therapeutic targets will have a similar impact on the metabolome of *S. plicata* and will cluster together in PLS-DA scores plot^10^. Hence, the action of a novel chemical lead can be inferred from its clustering in PLS-DA scores plot relative to drugs with defined biological targets. Similar statistical method was applied to predict *in vivo* mechanism of action to find therapeutic drug leads against *M. smegmatis* using NMR metabolomics^9^. Generally, if the chemical lead is separated from known compounds in the PLS-DA scores plot^9^, then this result would suggest a new mechanism of action and could be serve as potentially valuable new clinical biomarkers^15^. The method, using LC–MS and multivariate analysis, could be a useful tool for quality control issues and effectively utilized in drug discovery process.

### Structure elucidation of compounds

HPLC analysis of *S. plicata* CE is given in (Figure S1). Different retention times (RT) with various combinations of water and MeOH were used for the present HPLC method. Fraction SP-8 was chosen from the 60 HPLC fractions and purified further (Figure S4), which were analyzed by LC–MS to investigate the *m/z* of this purified fraction *m/z* 128.06. Using an extensive 1D and 2D NMR spectral data (Table S4), dihydro-5-methylpyrimidine – 2, 4 (1H, 3H)-dione (**1**) was tentatively identified from the fraction SP-8 with a molecular formula of C_5_H_6_N_2_O_2_ (NMR spectral was enclosed in Supplementary Information).
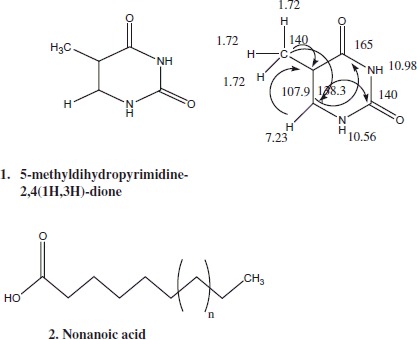


Further, the purified fractions (SP-28, SP-50, SP-53, SP-55) molecular weight, ^1^H-chemical shifts and 2D NMR spectroscopy was recorded on LC–MS and NMR spectrometry. Remarkably, all the fractions were identified as long-chain fatty acids and molecular weight of all the fractions was acquired using mass spectroscopy and given in Supplementary information (Figure S5–S6). Fatty acid, nonanoic acid was tentatively identified from the fraction SP-53 (NMR spectral data were enclosed in Supplementary information). Similarly, a modified octapeptide, plicatamide and their analogs have been isolated from the same ascidian *S. plicata* collected at San Diego Bay^28^. In this study, pyrimidine derivatives, nonanoic acid along with three long-chain fatty acid fractions were isolated in *S. plicata*. Initially, Wasylyk and Alam^29^ isolated diterpene styelidae from the same ascidian species collected in California. Anti-inflammatory heparin-like analogs dermatasulfate was isolated in *S. plicata* collected in Brazil coast^30^. Recently, six MNPs (e.g. tetillapyrone, nortetillapyrone, two nucleosides thymidine) were reported in *S. plicata* collected in China coast^31^.

### Antitumor activity of ascidian extracts

The crude extracts of ascidians have shown a lot of interest due to their higher antibacterial, antifungal and cytotoxicity activities^16^^,^^32^. The MeOH extracts of ascidians *S. plicata* and *A. mentula* exhibited significant cytotoxicity and strongly inhibited nitric oxide (NO) production against human embryonic kidney cells (HEK-293) at concentration 1 mg/ml as reported in the previous study^33^.

The effect of ascidian CE of *S. plicata and A. mentula* on proliferation of four tumor cell lines from different origin was tested using MTT assay. As reported in Table 1, IC_50_ values for *S. plicata* CE range from 41 to 125 μmol/l. In particular, the *S. plicata* showed the highest activity against HeLa cervical cancer (IC_50_ 41.15 μmol/l), HT29 colon cancer (IC_50_ 42 μmol/l) and HT1080 fibrosarcoma cells (IC_50_ 52 μmol/l), while MCF7 breast cancer cells were more resistant (IC_50_ 125 μmol/l). On the contrary, the response of the four cell lines to *A. mentula* CE was less heterogeneous IC_50_ values ranging from 39 to 58 (HeLa, IC_50_ 53 μmol/l; HT29, IC_50_ 39 μmol/l; MCF7, IC_50_ 58.3 μmol/l; and HT1080, IC_50_ 50 μmol/l). Similarly, Nurhayati et al.^34^ have reported modest anticancer activity of sponge *Cinachyrella* sp crude extracts against HeLa cells (IC_50_ 82.744 μg/ml).

**Table 1.** Antitumor activity of methanol extracts of ascidians in four tumor cell lines determined by MTT assay.

Sample	Tumor cell lines	IC_50_ (μmol/l)
*S. plicata*	HeLa	41.15
	HT29	42
	MCF-7	125
	HT1080	52
*A. mentula*	HeLa	53
	HT29	39
	MCF-7	58.3
	HT1080	50

**Figure 4.**
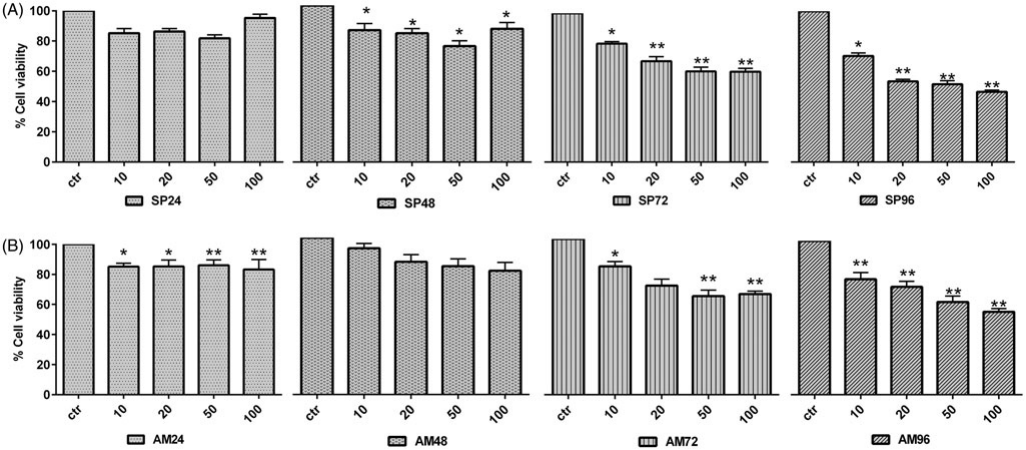
Cytotoxicity of ascidian crude extracts against HeLa cells. Analysis of cell viability by MTT assay using CE of *Styela plicata* (Figure 4(A)) and *Ascidia mentula* (Figure 4(B)) at concentrations ranging from 1 to 100 μM for 24–96 h. The results represents the mean ± SEM of replicates. One-way ANOVA were calculated between contol and treated cells. *p < 0.05, **p < 0.01.

Then, the response of HeLa cells to different time exposure (24–96 h) and concentrations (10–100 μmol/l) of *S. plicata* extracts was analyzed. As reported in Figure 4(A) a significant reduction of cell viability was observed at the longest time exposure (96 h) and at the highest concentrations used (20 and 50 μmol/l). Similarly, a significant reduction of cell viability was observed when HeLa cells were exposed to 10–50 μM

*A. mentula* extracts for 24–96 h (Figure 4(B)). Similar to our results Aiello et al.^35^ have reported two new sulfated metabolites, 3,7,11,15-tetramethyl-hexadecan-l,19-sodium disulfate and heneicosane-l,21-sodium disulfate from the same ascidian species with modest antiproliferative activity against human melanoma IGR-1 (IC_50_>110, 100 μg/ml) and murine monocyte/macrophage J774 (IC_50_>180, >170 μg/ml). The extract of *Didemnum ligulum* exhibited modest cytotoxicity against HCT-8 cells (IC_50_ 35.3 μg/ml) and *Didemnum psammatodes* fraction 1 showed strongest cytotoxicity against Molt-4 cells (IC_50_ 35.3 μg/ml)^36^^,^^37^.

### Antitumor activity of *S. plicata* fractions

**Figure 5.**
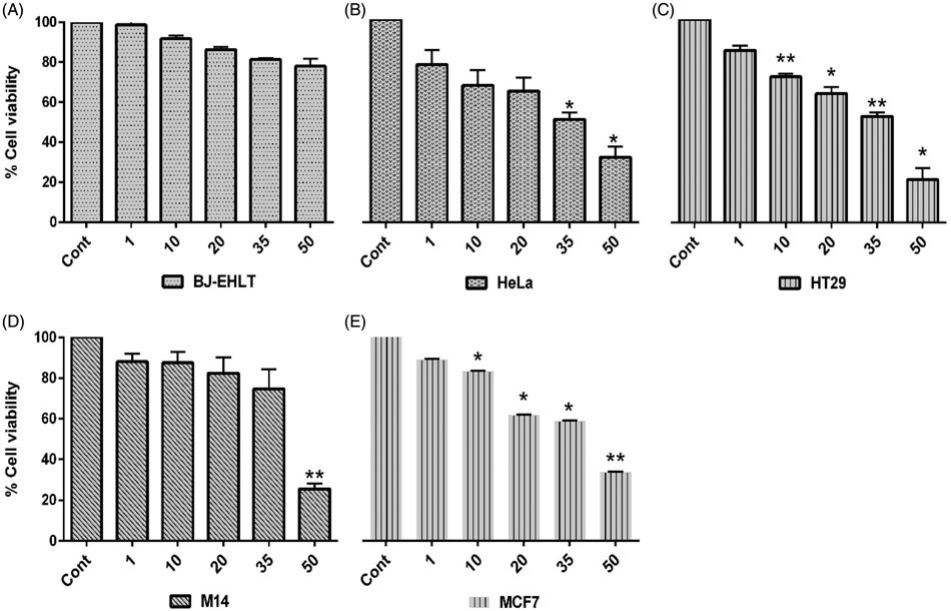
Dose-dependent cytotoxicity of fraction SP-50 of ascidian *S. plicata* against tumor and normal cells. Analysis of cell viability by MTT assay against normal BJ-EHLT (A) and four tumor (B–E) cell lines exposed to SP-50 at concentrations ranging from 1 to 50 μM for 24–96 h. The results represents the mean ± SEM of replicates. One-way ANOVA were calculated between contol and treated cells. *p < 0.05, **p < 0.01.

Table 2 shows the effect of five *S. plicata* fractions (SP-8, SP-28, SP-50, SP-53, SP-55) on HeLa cells determined by MTT assay. SP-8, SP-28, SP-53, SP-55 fractions showed modest antitumor activity against HeLa cells with IC_50_ values ranging from (37–46 μmol/l); on the other hand, the fraction SP-50 showed strongest growth inhibition (IC_50_ 33 μmol/l). Remarkably, SP-50 did not induce significant cell cytotoxicity in *BJ*-EHLT human fibroblasts (Figure 5). To study in depth the cytotoxicity effect of SP-50 the most effective fraction on tumor cells, we extended our analysis on a panel of tumor cell lines, including HT29, MCF7 breast cancer and M14 melanoma cells. As reported in Table 2, IC_50_ values ranging from 32 to 41 μmol/l were observed when HT29 (IC_50_ 31.66 μmol/l), MCF7 (37.29 μmol/l) and M14 (IC_50_ 41.05 μmol/l) cells were exposed to the fraction. A dose-dependent reduction of cell viability by fraction SP-50 was observed in all cell lines tested Figure 5. Accordingly with MTT assay results, our data showed a significant decrease in the number of viable cells in two cancer cells lines (HT-29 and HeLa) after 72 h of treatment with higher doses of SP-50 fraction (Figure S14).

**Table 2.** Antitumor activity of *S. plicata* fractions by MTT assay.

Compound/Fraction	Tumorcell lines	IC_50_ (μmol/l)
SP-8	HeLa	41.61
SP-28	–	46.33
SP-53	–	37.29
SP-55	–	38.2
SP-50	HT29	31.66
	MCF-7	37.29
	M14	41.05
	HeLa	33.27

Similar to this study, *Eudistoma vannamei* fraction 17 showed antiproliferative activity (IC_50_ value 1.85 μg/ml) and induced apoptosis and necrosis of human promyelocytic leukemia HL-60 cells^36^. Also, compounds, norteillapyrone and nucleosides thymidine were reported from the same ascidian with poor efficacy against *Artemia salina* (18.3%) at concentration 50 μg/ml^31^. Fatty acids metabolites could play an essential role in various biological functions, including cell proliferation and apoptosis induction. Moreover, inhibition of fatty acid oxidations blocks tumor growth of breast cancer^38^^,^^39^. The biological activity of fatty acids on cell proliferation is highly influenced by saturation of the molecule^39^.

### Apoptosis induction

**Figure 6.**
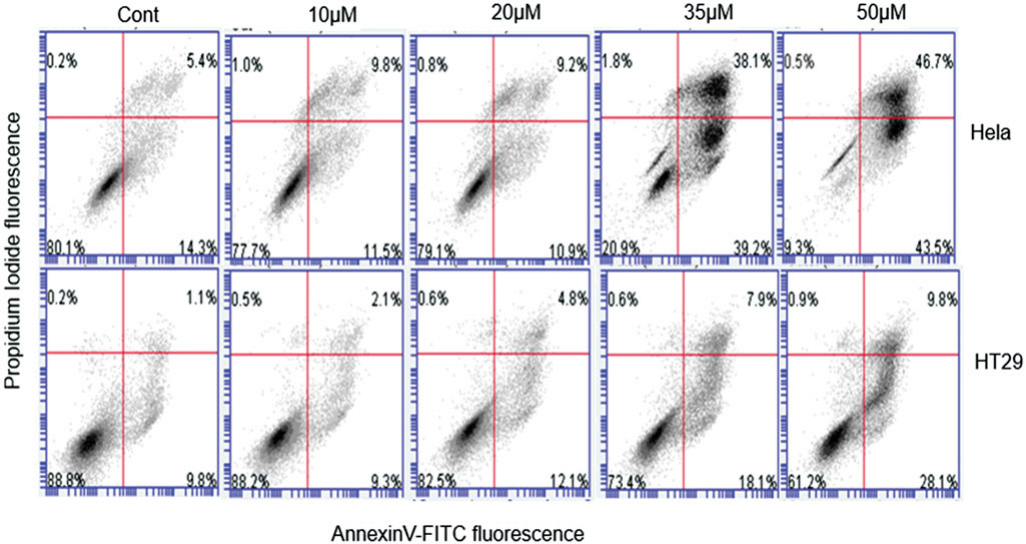
Flow cytometric analysis of apoptotic cells by annexin V/propidium iodide staining in HeLa and HT29 cells untreated or treated for 24 h with at the indicated concentrations. A representative experiment is shown. Annexin V+/PI − and annexin V+/PI+-stained cells were considered early apoptotic and late apoptotic or necrotic cells, respectively. Percentage of the different population of stained cells is shown.

Programed cell death or apoptosis is an important phenomenon in cancer cell death. Cell shrinkage, membrane blebbing, nuclear condensation and DNA fragmentation are the characteristic features of apoptosis^40^. To further determine whether the antiproliferative effects of *S. plicata* fractions were related to the induction of apoptosis and/or necrosis, SP-50-treated cells were analyzed with flow cytometry by using annexin V/propidium iodide (PI) staining that allows the discrimination of viable cells (annexin V+/PI+), early apoptotic (annexin V+/PI−), and late apoptotic or necrotic cells (annexin V+/PI+). As depicted in Figure 6, SP-50 induced apoptosis in a concentration-dependent manner in both the cell lines analyzed. In particular, 24 h of SP-50 with 50 μM treatment increased the percentage of both early (about 43% compared with untreated) and late apoptotic or necrotic events (about 47% compared with untreated) in HeLa cells.

In agreement with these results, a concentration-dependent increase in the Sub-G1 peak was observed after 72 h of treatment in both HeLa and HT29 treated cells when compared with untreated cells (Figures S12 and S13). Taken together, these data indicate that fraction SP-50 inhibited cell proliferation in both cell lines tested with higher effect when compared to other fractions of *S. plicata* and that the reduction in the cell viability was associated with the induction of apoptosis.

Similarly, induction of apoptosis has been reported with ecteinascidin-743 (ET-743) from *E. turbinata*^11^, aplidine from *A. albicans*^11^, CiEx, a partially purified compound from *Ciona intestinalis*^41^ and PI-8 fraction isolated from *Polyclinium indicum*^42^, and methyl ester mixtures of *D. psammatodes* exhibited strong apoptotic induction against HL-60 cells^37^. It is possible that *S. plicata* fraction SP-50 due to specificity of cancer cells, binds to DNA, inhibits cell division and triggers apoptosis similarly to ET-743^43^ and (dehydrodidemnin B) from *A. albicans*^44^. The marked arrest of cells in Sub-G1 phase and DNA fragmentation by the fraction SP-50 suggesting its possible use as anticancer drug. We can hypothesize a similar mechanism of action to the ascidian metabolites didemnin B and aplidine, which interfered with the synthesis of DNA and proteins and induced cell-cycle arrest^45^^,^^46^. The meridianin E from *A. meridianum*^47^ and lissoclinolide from *Lissoclinum patella*^48^ showed similar arrest of G2/M phase in the cell cycle of target cells.

## Conclusions

In conclusion, from an ecological perspective, the metabolome data suggest that secondary metabolites could apparently account chemical defense in ascidian *S. plicata* and rich resource of compounds with cytotoxic properties. LC–MS metabolomics of ascidian and PLS-DA resulted in distinct clustering patterns correlating with *in vitro* or *in vivo* biological activity of metabolites. The clustering of ascidian biomolecules relative to known biological activity could be used to discriminate the compounds based on pharmacological properties and classify the compounds with unknown biological activities. This study indicates that the presence of several metabolites in ascidian *S. plicata* crude extracts can be used effectively in pharmaceutical industries during drug discovery program. In this study, two biotoxin metabolite were identified in ascidian *S. plicata*; therefore, ascidian caught in Lake Faro not safe for human consumption. The results of cell-cycle analysis indicate that fraction SP-50-inhibited cell proliferation in both cell lines tested and that the reduction in the cell viability was associated with induction of apoptosis and/or necrosis, being SP-50 the strongest apoptosis inducer with highest growth inhibition against HeLa cells compared to other fractions of *S. plicata*. The outcomes from this present study suggest that *S. plicata* fraction SP-50 is a useful anticancer compound, which enhances a therapeutic efficacy; further studies are necessary to clarify this point. However, further studies of isolation pure compounds and structural identification of these active compounds present in *S. plicata* and molecular mechanism of pure compounds induced apoptosis are need to be discovered. The results of this study open new hope for discovering novel marine drugs from Mediterranean ascidian with potential therapeutic in the treatment of cervical and colon cancer disease.

## Supplementary Material

IENZ_1266344_Supplementary_Material.pdf
