# Supplementary material for: Metabolite profiling of ascidian Styela plicata using LC–MS with multivariate statistical analysis and their antitumor activity
Source: J Enzyme Inhib Med Chem. 2017 Feb 24;32(1):614–23. doi: 10.1080/14756366.2016.1266344 (PMC6010017; doi:10.1080/14756366.2016.1266344)
Supplement: IENZ_1266344_Supplementary_Material.pdf [file IENZ_A_1266344_SM2163.pdf]

Table S1. FTIR chemical characterization of ascidian crude extracts

Band position cm^{-1}	Group frequency	Functional group Assignments
STYELA PLICATA		
**3300-3500**	Amine N-H	Amide N-H stretch
**3400**	Hydroxy group O-H	O-H stretching frequencies of phenol
**2970-2860**	Alkane, Methyl ($-CH_3$)	Methyl C-H asym./sym. Stretch
**1615-1580**	C=C-C	Aromatic ring stretch
**1680-1620**	Alkene C=C	Alkenyl C=C stretch
**1420-1410**	Alkane/ Alkyl Methylene (CH_2)	Vinyl C-H out of plane
**1350-1000**	Acetylenic Compounds	C-C stretch (skeletal vibration)
**1290-1310**	Alkene C-H	Vinylidene C-H in plane bend
**1160-1170**		Symmetric ($-SO_2$ stretching vibration)
**1150-1000**	Aliphatic organohalogen C-F	Aliphatic fluoro compounds, CF stretch
**895-885**	C-H	Vinylidene C-H out of plane bend
ASCIDIA MENTULA		
**3300-3500**	Amine N-H	Amide N-H Stretch
**3320-3310**	Alkyne C-H	C-H bend
**2970-2860**	Alkane, Methyl ($-CH_3$)	Methyl C-H asym./sym. Stretch
**1615-1580**	C=C-C	Aromatic ring stretch
**1660-1500**	NO_2 Nitro compounds	Asymmetrical stretch
**1470-1430**	Methyl ($-CH_3$),aliphatic (alkane/alkyl)	Methyl CH asym./sym. Bend
	(saturated aliphatic group)	
**1240-1210**	Alkene group C-H	Vinylidene CH in-plane bend
**1110**	C-O	Secondary alcohol, C-O stretch
**995-985**	Alkene C-H	Vinyl CH out-of-plane bend

Table S2. Relative intensities of identified metabolites in *S. plicata* crude extracts

S. NO	RT	M/Z	METABOLITES	MOL FORMULA	CHEMICAL GROUP	METLIN	HMDB	KEGG/LMID	ADDUCT	ADDUCT M/Z	ERR OM/Z	>50 PPM	BIOLOGICAL ACTIVITY
**1**	1.15	105.48	Metaxalone	$C_{12}H_{15}NO_3$	Aromatic ether	43910		C07934	-	105.48	18	-	Skeletal Muscle Relaxants
**2**	1.16	107.05	3-(2-(methylamino)ethyl)-1H-indol-5-ol	$C_{11}H_{14}N_2O$	Tryptamine alkaloid.	24110		C06212	$[M+H+Na]^{2+}$	107.05	2	-	Tryptophan metabolism
**3**	1.15	107.12	1-Methylpiperazine	$C_5H_{12}N_2$	Synthetic carbamyl derivative of piperazine	2008			$[M+Li]^+$	107.11	0	-	Parasitic diseases
**4**	1.15	130.36	Repenol	$C_{18}H_{12}O_{10}$	Flavanoids	48089		LMPK12060080	$[M+3H]^{3+}$	130.35	7	-	
**5**	1.29	138.36	Decarbamoylgonyautoxin 1	$C_9H_{16}N_6O_8S$	Decarbamoyl derivatives	73507		C20022	$[M+2Na+H]^{3+}$	138.35	10	-	Paralytic shellfish poisoning
**6**	1.09	140.43	Simvastatin	$C_{25}H_{38}O_5$	Hydroxymethylglutaryl-coenzyme A	2443		C07262	[M+3H]3+	140.43	8	-	Inhibitor of cholesterol synthesis
**7**	10.09	149.38	2-([4-(2-Chlorophenyl)-3-ethoxycarbonyl-6-hydroxymethyl-2-picolinyl]methoxyacetic acid	$C_{19}H_{18}ClNO_8$	Methoxy aceticacid	65347	HMDB00696	C00073	[M+2Na+H]3+	423.07	9	-	
**8**	1.23	160.52	Flumazenil acid	$C_{13}H_{10}FN_3O_3$	Ethyl ester	2726		C07825	$[M+2Na]^{2+}$	160.52	3	-	$GABA_A$ receptor antagonist
**9**	8.93	167.52	Isocarbophos	$C_{11}H_{16}NO_4PS$		263529			$[M+2Na]^{2+}$	167.52	6		Ecotoxicity
**10**	5.52	171.55	Benzamil	$C_{13}H_{14}ClN_7O$	Carboxamide	69682		C13751	$[M+H+Na]^{2+}$	171.55	1		

**11**	1.30	184.58	Pefurazoate	$C_{18}H_{23}N_3O_4$	Imidazole	72286		C18480	$[M+H+Na]^{2+}$	184.58	1		Anti- fungal activity
**12**	5.24	193.59	Retrofractamide D	$C_{21}H_{27}NO_3$	Alkaloid	89282	HMDB33450		$[M+2Na]^{2+}$	193.58	1		Cell signalling
**13**	6.22	197.56	2-(Biaryl)carbapenems	$C_{21}H_{19}NO_4$	Biphenyls	69256		C12019	$[M+2Na]^{2+}$	197.55	10		
**14**	6.37	197.62	Tyr Leu Val	$C_{20}H_{31}N_3O_5$	Amino acids	15886			$[M+2H]^{2+}$	197.62	2		
**15**	1.30	198.69	Oxidized Watasenia luciferin	$C_{25}H_{21}N_3O_9S_2$	Pyrazines	66073		C03888	$[M+2H+Na]^{3+}$	198.69	9		
**16**	1.23	207.57	Proacaciberin	$C_{16}H_{25}NO_{10}$	Amino acid	66916		C08337	$[M+H+Na]^{2+}$	207.57	5		
**17**	5.94	212.67	Linoleyl carnitine	$C_{25}H_{45}NO_4$	Fatty acids	58418	HMDB06469		$[M+2H]^{2+}$	212.67	12		Lipid catabolism
**18**	9.07	213.62	16-phenoxy tetranor PGF2α methyl amide	$C_{23}H_{33}NO_5$	Methyl amide	64705			$[M+H+Na]^{2+}$	213.62	3		
**19**	5.94	219.00	Lyngbyatoxin	$C_{27}H_{39}N_3O_2$	Alkaloid	71051		C15720	$[M+2H]^{2+}$	219.65	16	*	Marine Biotoxin
**20**	6.29	223.63	Mivacurium Metabolite	$C_{25}H_{35}NO_6$	Isomeric	1328			$[M+2H]^{2+}$	223.63	2		Muscle relaxant Metabolite
**21**	8.14	225.52	Difenoconazole	$C_{19}H_{17}Cl_2N_3O_3$	Aromatic ether	72265		C18459	$[M+2Na]^{2+}$	225.52	6		Anti- funal activity
**22**	8.50	239.57	8-Methylthiooctyl glucosinolate	$C_{16}H_{31}NO_9S_3$	Thioethers	71613		C17254	$[M+2H]^{2+}$	239.57	1	*	
**23**	1.51	247.56	Glucohirsutin	$C_{16}H_{31}NO_{10}S_3$	Amino acid	71617		C17271	$[M+2H]^{2+}$	247.56	0		
**24**	9.53	249.71	Loropetalin D	$C_{35}H_{28}O_{19}$	Flavanoids	50352		LMPK12111936	$[M-3H]^{3-}$	249.70	0		
**25**	3.34	252.67	Pederin	$C_{25}H_{45}NO_9$	Alkaloid	71091		C15760	$[M+2H]^{2+}$	252.66	11		
**26**	9.07	253.61	Pentazocine glucuronide	$C_{25}H_{35}NO_7$	Alkaloid	1752			$[M+2Na]^{2+}$	253.6	0		Opioid agonist-antagonist,
**27**	4.52	261.67	Oleyloxyethyl Phosphorylcholine	$C_{25}H_{52}NO_5P$	Amide	63061			$[M+2Na]^{2+}$	261.67	10		inhibitor of PLA2
**28**	2.78	268.66	Maraviroc	$C_{29}H_{41}F_2N_5O$	Alkaloid	85537	HMDB15584		$[M+H+Na]^{2+}$	268.6	0		Anti- HIV
**29**	9.54	269.73	1,1'-(1,4-Dihydro-4-nonyl-3,5-pyridinediyl)bis[1-	$C_{34}H_{61}NO_2$	Alkaloid	90798	HMDB35517		$[M+H+Na]^{2+}$	269.73	1		Nutrient

			decanone]										
**30**	9.65	277.67	LysoPE(0:0/24:6(6Z,9Z,12Z,15Z,18Z,21Z))	$C_{29}H_{48}NO_7P$	Lysophospholipid	62287	HMDB11499		$[M+2H]^{2+}$	277.6	8		Cell signalling
**31**	1.09	280.57	18:1-Glc-Stigmasterol	$C_{53}H_{90}O_7$	Sterol lipids	103450		LMST01040229	$[M+3H]^{3+}$	280.56	5		
**32**	1.09	280.63	Candoxatril	$C_{29}H_{41}NO_7$	Indane	85408	HMDB14754		$[M+2Na]^{2+}$	280.63	19	*	Pro drug, Protease Inhibitors
**33**	10.09	281.76	Cer(d18:0/16:0)	$C_{34}H_{69}NO_3$	Sphingolipids	41565		LMSP02020001	$[M+H+Na]^{2+}$	281.76	0		
**34**	5.18	282.71	LysoPE(24:1(15Z)/0:0)	$C_{29}H_{58}NO_7P$	Lysophospholipid	62312	HMDB11528		$[M+2H]^{2+}$	282.70	7		Membrane integrity/stability
**35**	1.16	285.79	Betavulgaroside VII	$C_{41}H_{62}O_{16}$	Carboxylic acid	89262	HMDB33427		$[M+2Na+H]^{3+}$	285.80	12	*	Nutrient
**36**	8.83	286.80	1,1-Dibromo-1-chloro-2-propanone	$C_3H_3Br_2ClO$	Ketones	94722	HMDB40187		$[M+K]^{+}$	286.78	48	*	Nutrient
**37**	10.05	291.65	N3'-Acetylapramycin	$C_{23}H_{43}N_5O_{12}$	Macrolids	65851		C02856	$[M+2H]^{2+}$	291.65	9		
**38**	9.54	300.85	3,5-Dibromo-4-hydroxyphenylpyruvate	$C_9H_6Br_2O_4$	2-oxo monocarboxylic acid	66153		C04285	$[M+H-2H2O]^{+}$	300.85	16		
**39**	9.11	300.85	2,3,4,5,6-Pentachlorobenzyl alcohol	$C_7H_3Cl_5O$	Benzenoids	94952	HMDB40450		$[M+Na]^{+}$	300.85	22		Effect in foliar-blast disease of rice
**40**	7.46	300.85	Unknown										
**41**	2.43	314.64	Tri-N-acetylchitotriose	$C_{24}H_{41}N_3O_{16}$	Acetoamide	58520	HMDB06698		$[M+2H]^{2+}$	314.63	20	*	
**42**	10.60	319.68	PC(16:0/5:0(CHO))	$C_{29}H_{56}NO_9P$	Glycerophospholipids	82378		LMGP20010005	$[M+2Na]^{2+}$	319.6	9		
**43**	8.57	328.81	Cer(t20:0/20:0(2OH))	$C_{40}H_{81}NO_5$	Sphingolipids	103035		LMSP02030021	$[M+2H]^{2+}$	328.8	12		
**44**	10.89	337.75	Lepidimoic acid	$C_{36}H_{54}O_{30}$	Carboxylic acid	95529	HMDB41096		$[M+2Na+H]^{3+}$	337.75	0		
**45**	11.71	340.84	Lanosol	$C_7H_6Br_2O_3$	Isomeric	71557		C17098	$[M+2Na-H]^{+}$	340.84	4		

**46**	4.04	345.63	Prednimustine	$C_{35}H_{45}Cl_2NO_6$	Corticosteroid hormone	73195		C19512	$[M+2Na]^{2+}$	345.62	13		Anti neoplastic agensts
**47**	9.65	349.66	A 80987	$C_{37}H_{43}N_5O_6$	Amino acid amide	71004		C15661	$[M+2Na]^{2+}$	349.64	14		
**48**	11.01	351.73	Anthenoside A	$C_{38}H_{65}NO_9$	Sterol Lipids	84941		LMST05050021	$[M+H+Na]^{2+}$	351.73	7		
**49**	11.29	361.81	Unknown										
**50**	11.47	361.81	Unknown										
**51**	9.81	367.73	Microcolin C	$C_{37}H_{63}N_5O_7$	Lipopeptide	65421			$[M+2Na]^{2+}$	367.73	14		Antimitotic
**52**	11.41	367.79	Betaine lipids	$C_{42}H_{81}NO_7$	Glycerolipids	46617		LMGL00000125	$[M+H+Na]^{2+}$	367.79	13		Reported in algae
**53**	9.87	370.75	PS(P-16:0/18:4(6Z,9Z,12Z,15Z))	$C_{40}H_{70}NO_9P$	Glycerophospholipids	78755		LMGP03030015	$[M+2H]^{2+}$	370.74	19	*	
**54**	11.80	407.79	PC(18:1(9Z)/P-18:1(9Z))	$C_{44}H_{84}NO_7P$	Phosphatidylcholine	59575	HMDB08129	C00157	$[M+2Na]^{2+}$	407.79	3	*	
**55**	2.78	409.49	4,8-Dimethylheptacosane	$C_{29}H_{60}$	Fatty acids	97821		LMFA11000427	$[M+2Na]^{2+}$	409.49	14		
**56**	10.30	411.69	Unknown										
**57**	10.27	420.76	PS(P-18:0/20:4(5Z,8Z,11Z,14Z))	$C_{44}H_{78}NO_9P$	Glycerophospholipids	78830		LMGP03030090	$[M+2Na]^{2+}$	420.75	2		
**58**	7.46	447.59	Pyrrophenone	$C_{49}H_{37}F_2N_3O_5S_2$	Pyrrolidines	45521			$[M+2Na]^{2+}$	447.59	14		Inhibitor of of cytosolic PLA2
**59**	9.80	457.60	S-2-Octenoyl CoA	$C_{29}H_{48}N_7O_{17}P_3S$	Fatty Acyls	58140	HMDB02992		$[M+H+Na]^{2+}$	457.60	8		Lipid biosynthesis
**60**	11.77	482.61	Fucalpha1-2Galbeta1-4GlcNAcbeta1-3Galbeta1-4Glcbeta-Cer(d18:1/18:0)	$C_{68}H_{124}N_2O_{27}$	Sphingolipids	54954		LMSP0505AD02	$[M+2Na+H]^{3+}$	482.60	0		
**61**	10.76	483.74	Kabiramide B	$C_{47}H_{69}N_5O_{14}$	Alkaloid	65483			-	483.73	26		

**62**	10.76	504.71	Tetrabromodiphenyl ethers	$C_{12}H_6Br_4O$	Ethers	92390	HMDB37520		$[M+Na]^+$	504.70	18		Persistent environmental pollutants
**63**	10.35	507.86	Unknown										
**64**	10.89	534.76	Cyclolinopeptide I	$C_{55}H_{73}N_9O_9S_2$	Aminoacids	91633	HMDB36552		$[M+2H]^{2+}$	534.75	0		Nutrient
**65**	10.83	541.68	Icosenoyl-CoA	$C_{41}H_{72}N_7O_{17}P_3S$	Fatty acids	63374		C16530	$[M+H+Na]^{2+}$	541.69	24	*	Fatty acids metabolism
**66**	10.44	551.76	Crossbyanol D	$C_{31}H_{17}Br_7O_8S$	Poluphenyl ether	65380			$[M+2H]^{2+}$	550.76	13	*	
**67**	10.83	562.72	Tetrabromobisphenol A	$C_{15}H_{12}Br_4O_2$	bromobisphenol	69598		C13620	$[M+Na]^+$	551.75	48	*	Microbial metabolism
**68**	10.73	595.78	Unknown										
**69**	10.35	775.72	TG(13:0/15:1(9Z)/18:0)[iso6]	$C_{49}H_{92}O_6$	Glycerolipids	99619		LMGL03013764	$[M-H]^-$	562.74	48	*	
**70**	10.66	859.74	TG(15:0/18:4(6Z,9Z,12Z,15Z)/19:1(9Z))[iso6]	$C_{55}H_{96}O_6$	Glycerolipids	101069		LMGL03015221	$[M+Li]^+$	775.68	0		
**71**	10.77	1365.91	Galalpha1-3(GalNAcbeta1-4)Galbeta1-4Glcbeta-Cer(d18:1/26:1(17Z))	$C_{70}H_{128}N_2O_{23}$	Sphingolipids	55720		LMSP0505DO08	$[M+H]^+$	859.73	10		

Table S3. Relative intensities of identified metabolites in *A. mentula* crude extracts

S.NO	RT	M/Z	METABOLITES	MOL FORMULA	CHE GROUP	METLIN	HMDB	KEGG/LMID	ADDUCT	ADDUCT M/Z	ERROR M/Z
**1**	11.91	117.07	2-Methyl-1,3-cyclohexadiene	C_7H_{10}	Terpinene	88419	HMDB32395		$[M+Na]^+$	117.07	4
**2**	1.23	119.15	n-hexane	C_6H_{14}	Alkane	36775		LMFA11000007	$[M+CH3OH+H]^+$	119.14	24
**3**	1.17	146.42	Lespedezaflavanone G	$C_{27}H_{32}O_5$	Flavonoids	52829		LMPK12140345	$[M+3H]^{3+}$	146.42	0
**4**	3.71	152.40	S-Furanopetasitin	$C_{24}H_{32}O_5S$	Prenol lipids	91287	HMDB36131		$[M+2H+Na]^{3+}$	152.4	1
**5**	6.51	174.32	Isorhamnetin 3,4'-di-O-sulfate	$C_{16}H_{12}O_{13}S_2$	Flavonoids	50815		LMPK12112399	$[M+2Na+H]^{3+}$	174.31	12
**6**	7.12	174.51	Monocyclic botryococcane	$C_{34}H_{68}$	Lipids	53759		LMPR0106030003	$[M+2Na+H]^{3+}$	174.51	0
**7**	7.42	185.34	Dicloxacillin sodium	$C_{19}H_{18}Cl_2N_3NaO_6S$	Amide	69685		C13756	$[M+2Na+H]^{3+}$	185.33	16
**8**	6.44	197.37	ADP-glucose	$C_{16}H_{25}N_5O_{15}P_2$	ADP α-D-glucoside	63203		C00498	$[M+3H]^{3+}$	197.37	0
**9**	4.18	246.43	Urdamycin G	$C_{37}H_{46}O_{14}$	anthraquinone	63803		C12414	$[M+2H+Na]^{3+}$	246.43	3
**10**	2.85	268.41	Brassicoside	$C_{34}H_{42}O_{22}$	Flavonoids	86296	HMDB29480		$[M+3H]^{3+}$	268.41	10
**11**	10.66	279.50	2,5-Diaminopyrimidine nucleoside triphosphate	$C_9H_{18}N_5O_{14}P_3$	Pyrimidone	63908		C05923	$[M+2Na]^{2+}$	279.49	9
**12**	10.66	319.49	Unknown								
**13**	10.95	340.78	1,1-Dibromo-3-iodo-2-propanone	$C_3H_3Br_2IO$	Ketones	94724	HMDB40189		$[M+H]^+$	340.76	24
**14**	10.59	351.48	PIP2[3',5'](17:0/20:4)	$C_{46}H_{92}N_3O_{19}P_3$	Glycerophospholipids	40896		LMGP08010002	$[M+2H+Na]^{3+}$	351.48	7
**15**	10.30	384.61	Cardiolipin (CDN)	$C_{58}H_{120}O_{17}P_2$	phospholipids	4169			$[M+3H]^{3+}$	384.60	7
**16**	9.81	400.54	3-O-(Xylb1-3Glcb1-2(Xylb1-3)Glcb1-4Galb)-	$C_{56}H_{94}O_{27}$	Sterol lipids	84226		LMST01080075	$[M+3H]^{3+}$	400.54	9

			(25R)-5alpha-spirostan-3beta-ol								
**17**	11.00	519.64	(S)-3-Hydroxytetradecanoyl-CoA	$C_{35}H_{62}N_7O_{18}P_3S$	Fatty acyl thioesters	58187	HMDB03934	C05260	$[M+2Na]^{2+}$	519.64	6
**18**	0.88	133.00	1-Isothiocyanato-2-(methylthio)ethane	$C_4H_7NS_2$	Thioether	93133	HMDB38442			133.24	0
**19**	1.44	144.27	2-Methyl-5-(methylthio)thiophene	$C_6H_8S_2$	Alkyl thioether	94788	HMDB40259			144.01	0
**20**	4.26	246.30	Milnacipran	$C_{15}H_{22}N_2O$	Benzenoids	85546	HMDB15602			246.17	0
**20**	8.33	509.87	heneicosane-1,21 sodium disulfate	$C_{21}H_{42}O_8S_2Na$	Alkyl sulfates				$[M-NaSO_3^{-}H]^{+}$	406.40	0
**21**	0.81	119.40	Hydroxyethyl glycine	$C_4H_9NO_3$	Amino acids		HMDB61148			119.06	0

Table S4. Chemical shift NMR Data for Fraction SP-8 in DMSO –d_6 (1H 600 MHz), ESI-HRMS (Mariner) spectrum showed one signal at *m/z* 129.

**Proton**	**Carbon/HSQC**	**COSY**	**HMBC**
10.98 (1H d)	165	8.98	
10.54 (1H, S)			140
8.98 (1H d	145.4	8.11	47.83
J' 6.0		8.97	127.27
			143.3
8.12 (1H, d-d	126.92	8.11	127.63
J' 7.2		8.5	143.3
		8.9	
7.23 (3H, S	137.29	1.73	11.75
		7.2	151.2
		10.54	164.66
4.34 (2H, S	47.61	4.32	145.3
3.22 (1H, S	52.93	3.3	53.19
			62.45
1.72 (8H, S	11.25	1.73	107.53
		7.22	137.62
			164.89
1.23 (2H, d	20.2	1.23	
		3.97	

Table S5. Chemical shift NMR Data for Fraction 50 in DMSO –d_6 (1H 600 MHz), ESI-HRMS (Mariner) spectrum showed one signal at *m/z* 390.

**Proton**	**Carbon/HSQC**	**Cosy**	**gDcosy**	**HSQAD Toxy**	**HMBC**
5.32	129	1.97	1.97	25.84	25.66
		2.7	2.7	129	
		5.3	5.3		
3.57	50.67	3.57	3.57	50.25	173.7
2.81	24.8	5.3	5.3	25.8	129
		2.7	2.7		
2.71	24.8	5.3	5.3	24.8	24.8
2.27	33.03	1.47	33.7	33	28.1
			24.8		173.7
2.17	26.7	1.47	1.47	24	24
	33	2.27	2.27	28	28
	39.7			33	173
1.98	26.39	1.23	1.23	26.25	28.5
		5.3	5.3	28.15	129
				129	
1.47	24.35	1.2	0.84	24	28
		2.27	1.2	28.6	33
			2.27	33	173
1.23	21.44	0.85	0.85	14	24
	14	1.23	1.2	24	28
	24	1.47	1.47	26	31
	26.7	1.98	1.98	28	
	28.3	2.17		33	
	33	2.27		129	
	39.7				
	90				
	97				
	160				
0.85	14	1.23	1.23	14	21.7
	21	1.47	1.47	28	28
	26			31	31
					39

Table S6. Chemical shift NMR Data for Fraction SP-53 in $CDCl_3$ (^1H 600 MHz), ESI-HRMS (Mariner) spectrum showed one signal at *m/z* 159

Proton	HSQC	Cosy	HMBC
7.701	129.9	7.5, 1.3	
7.517	130.8	1.3	
5.335	129.28	2.1	27.57
4.196	68.461	1.7	25.37
3.775	64.15	1.7	51
3.65	51.814	3.6	175.105
3.462	42.56	1.6	72.21, 26
2.29	34.078	1.6	180, 174
1.997	32.8	1.34, 1.6	130.55, 29
1.617	25.514	2.3, 1.6,1.3	180, 175, 29
1.248	29	1.2	29.1
0.912	14.1		14.1
0.118	0.34	0.12	

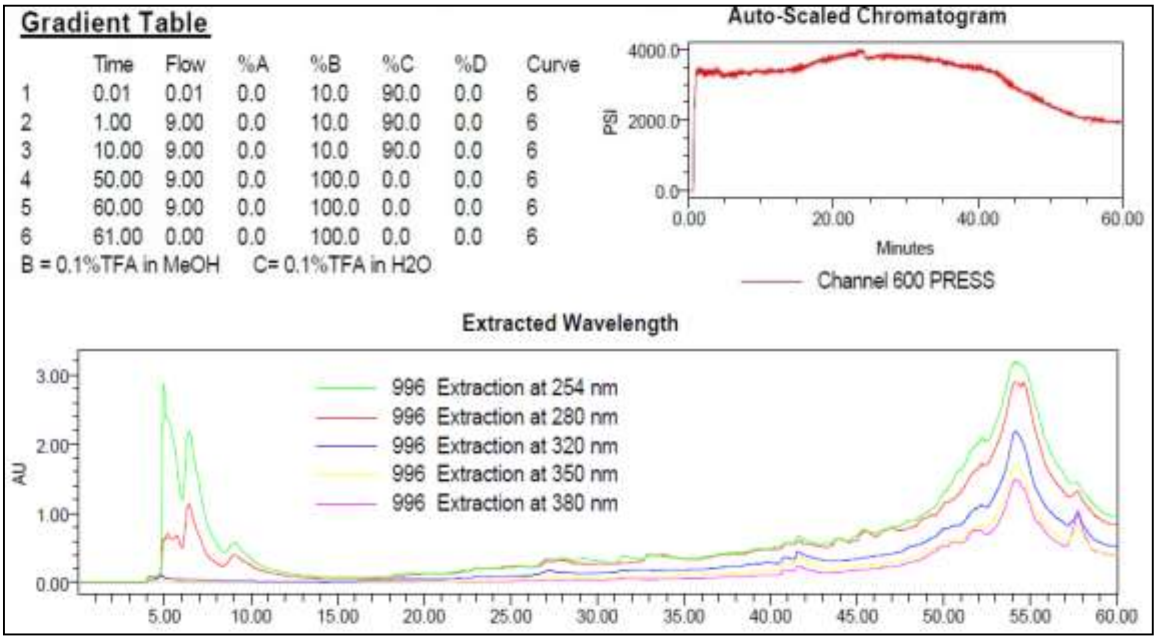

Figure S1. HPLC analysis of *S. plicata* crude extracts

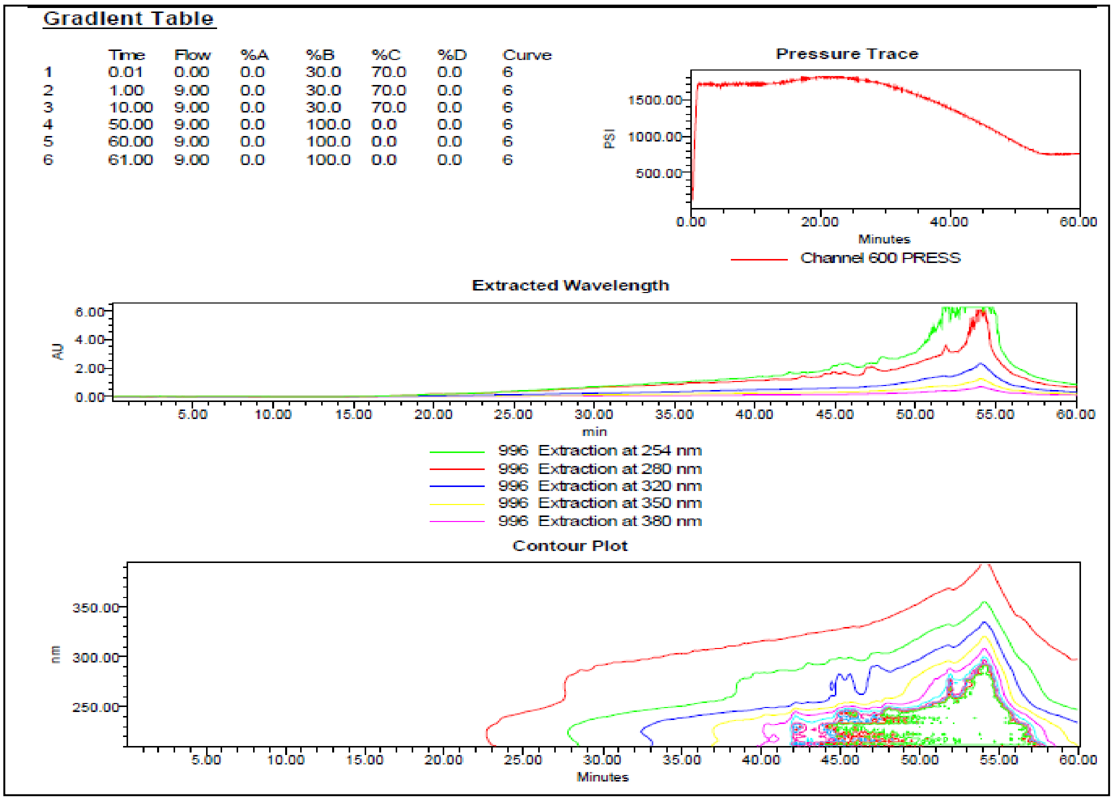

Figure S2. HPLC separation of *S. plicata* fraction SP-50, 53, 55

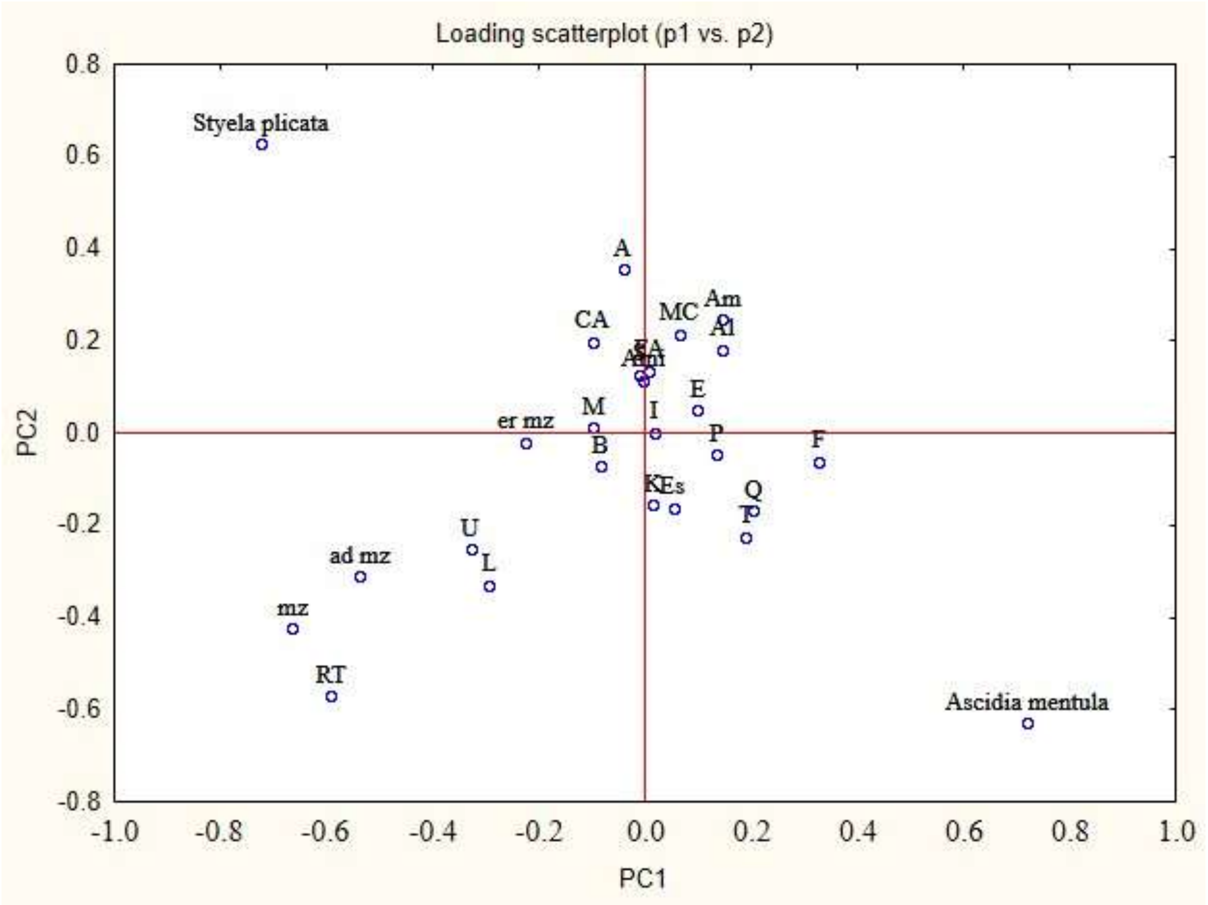

Figure S3. PCA loading score plot of S. plicata and A. mentual LC-MS spectral varaibles

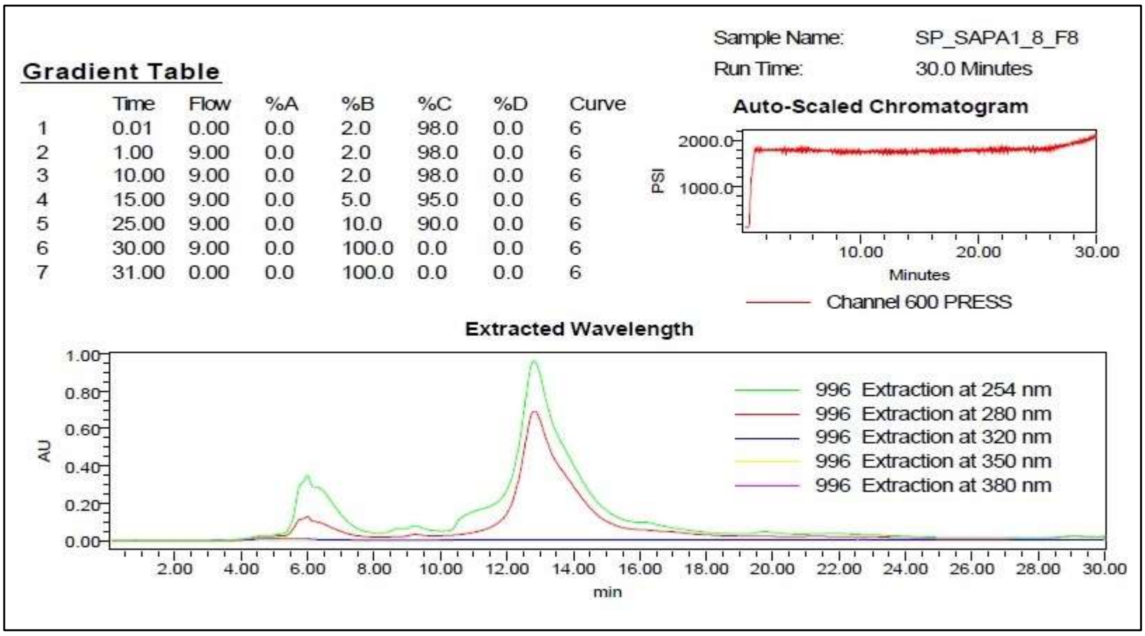

Figure S4. HPLC separation of *S. plicata* fraction SP-8

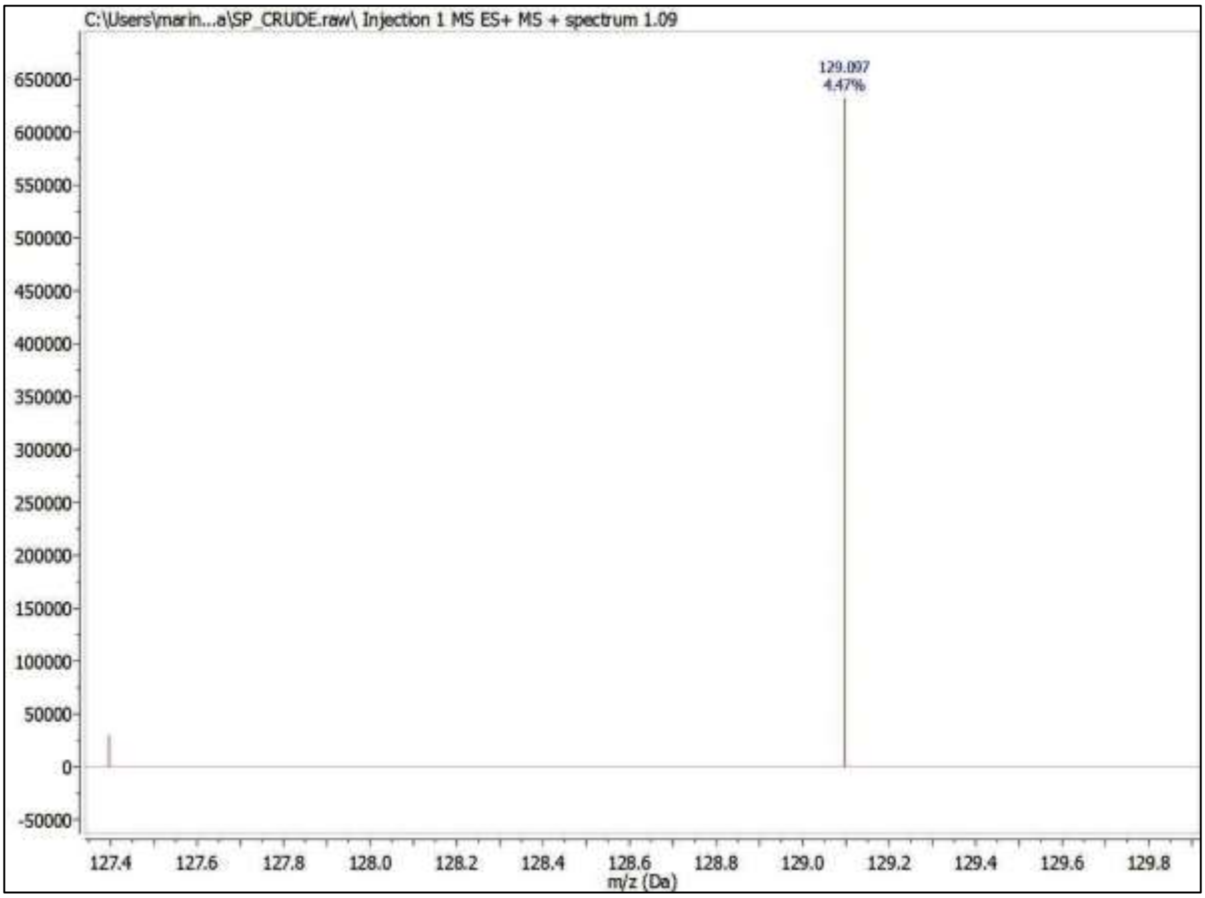

Figure S5. Molecular weight of fraction SP-8 of *Styela plicata*

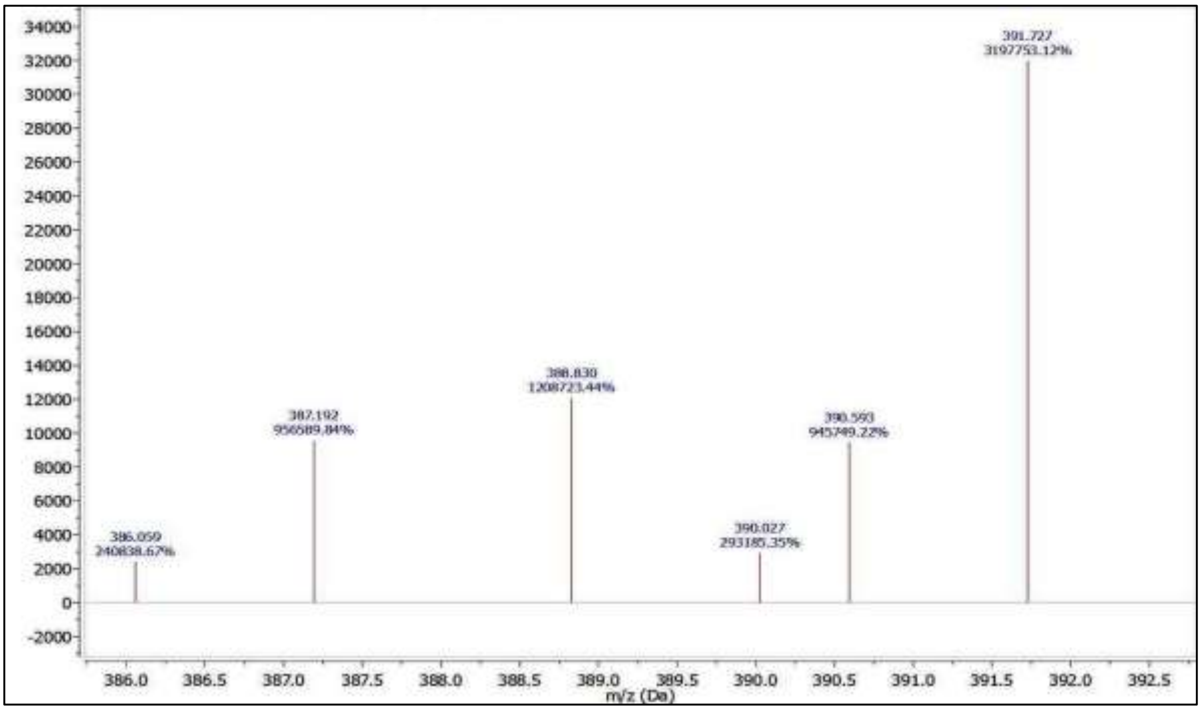

Figure S5. Molecular weight of fraction SP-50 of *Styela plicata*

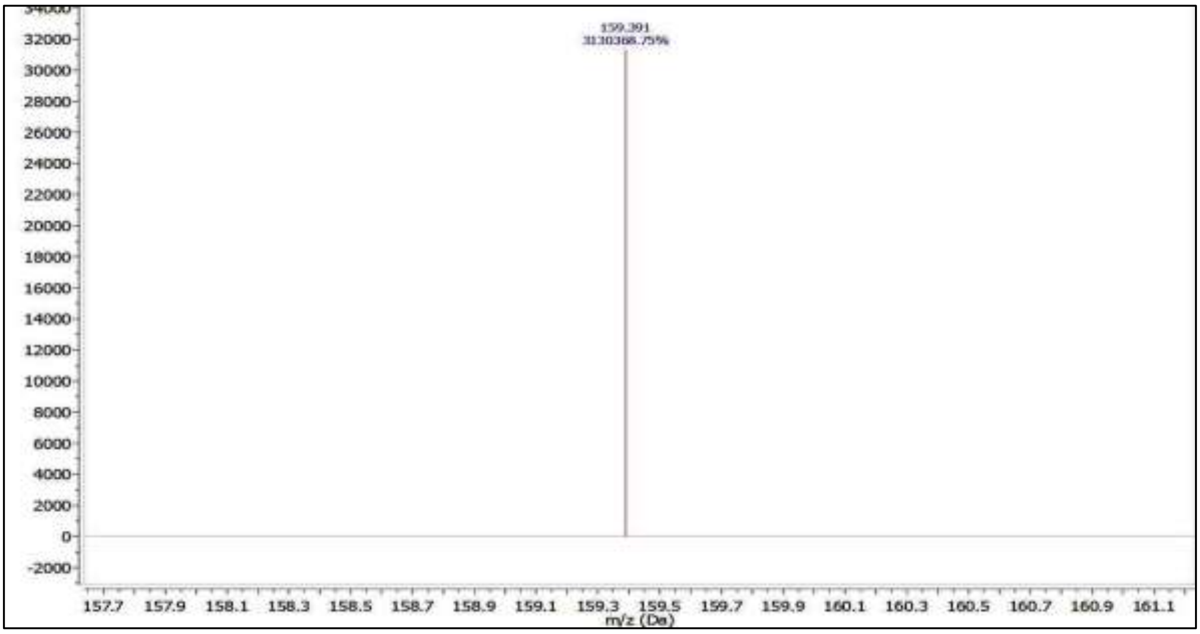

Figure S6. Molecular weight of Nonanoic acid (Fraction SP-53) of *S. plicata*

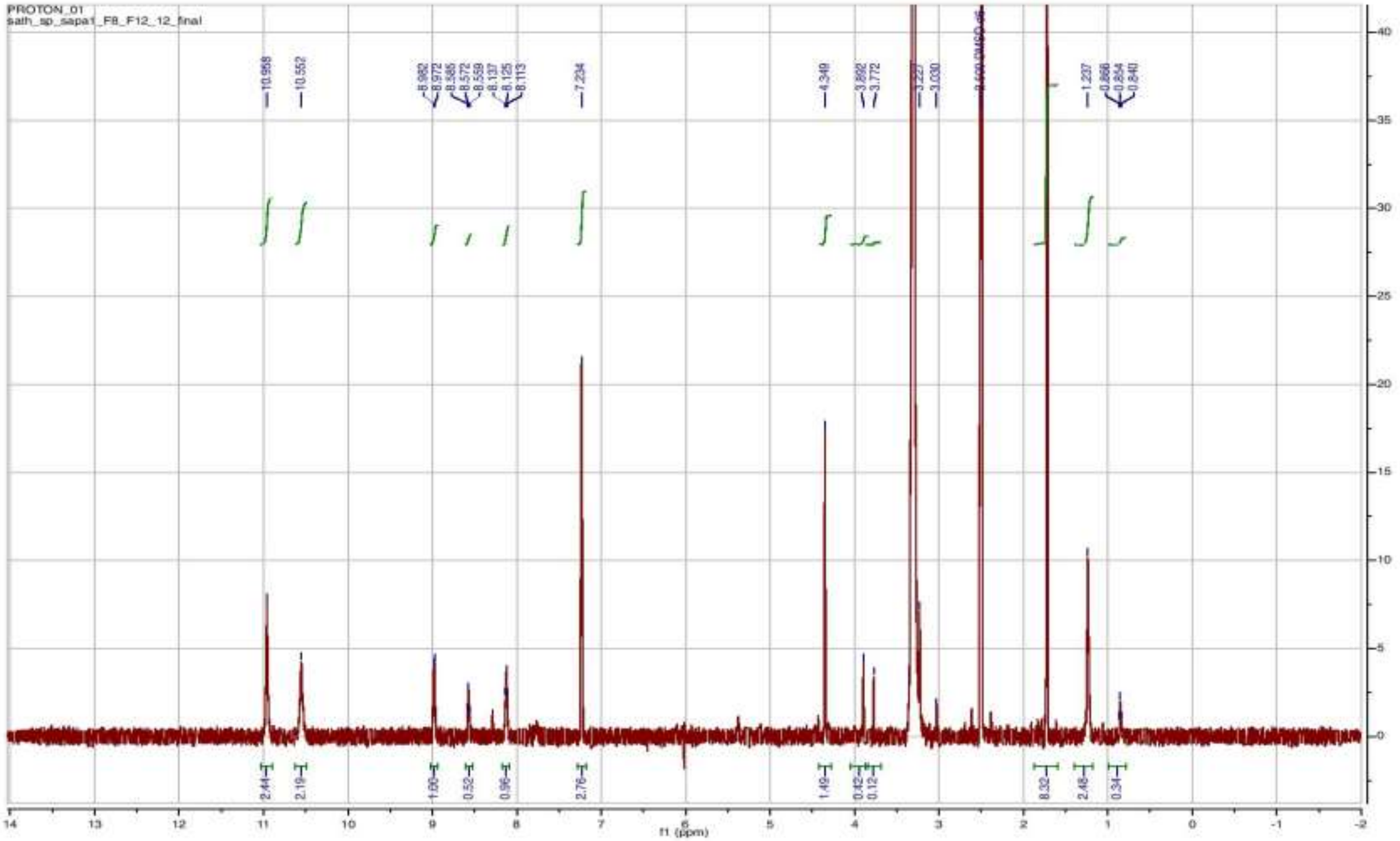

Figure S7a. **[1]HMR of *S. plicata* fraction SP-8 in DMSO- *d*6**

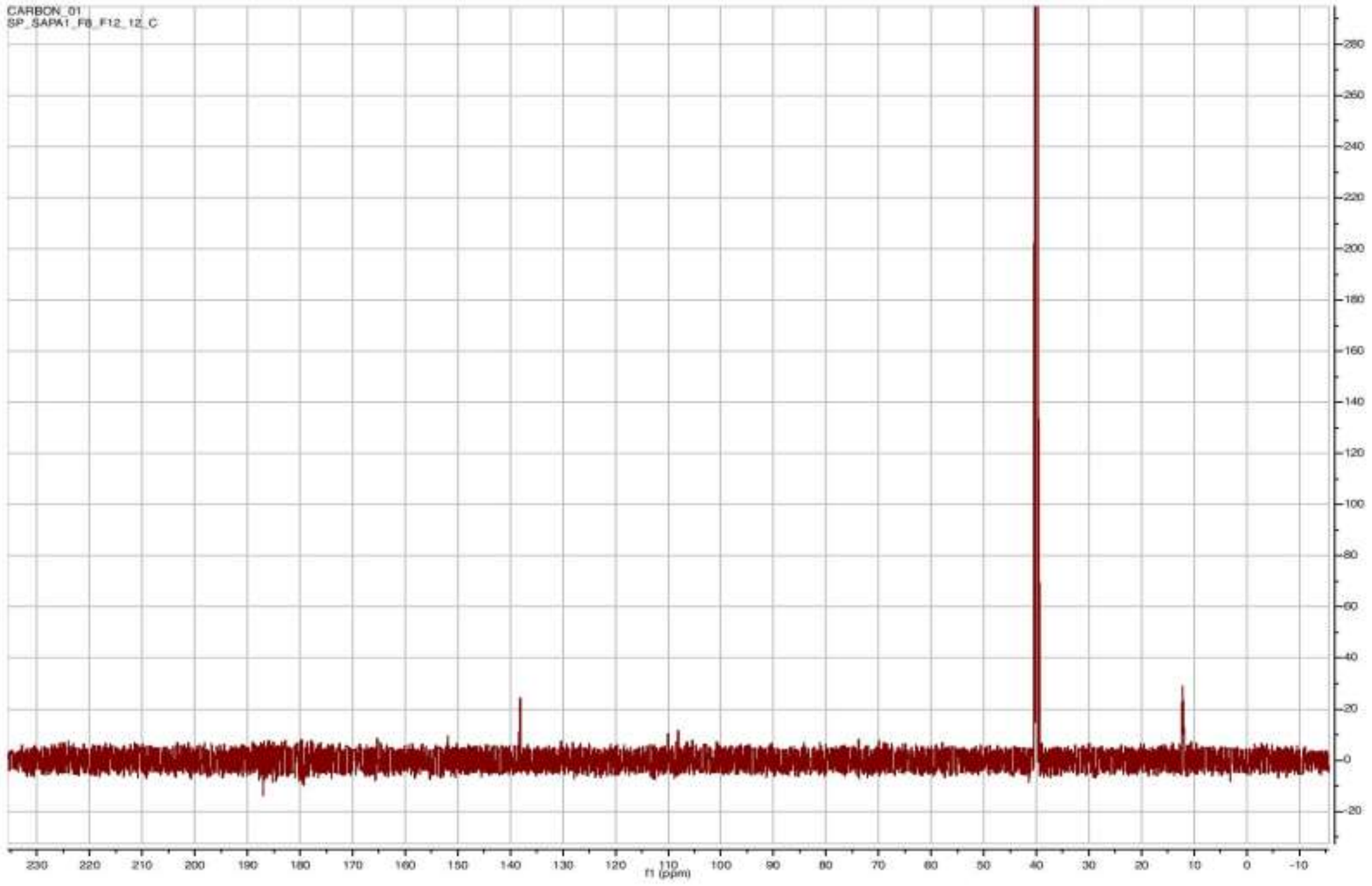

Figure S7b. 13C-NMR of *S. plicata* fraction SP-8 in DMSO- *d*6

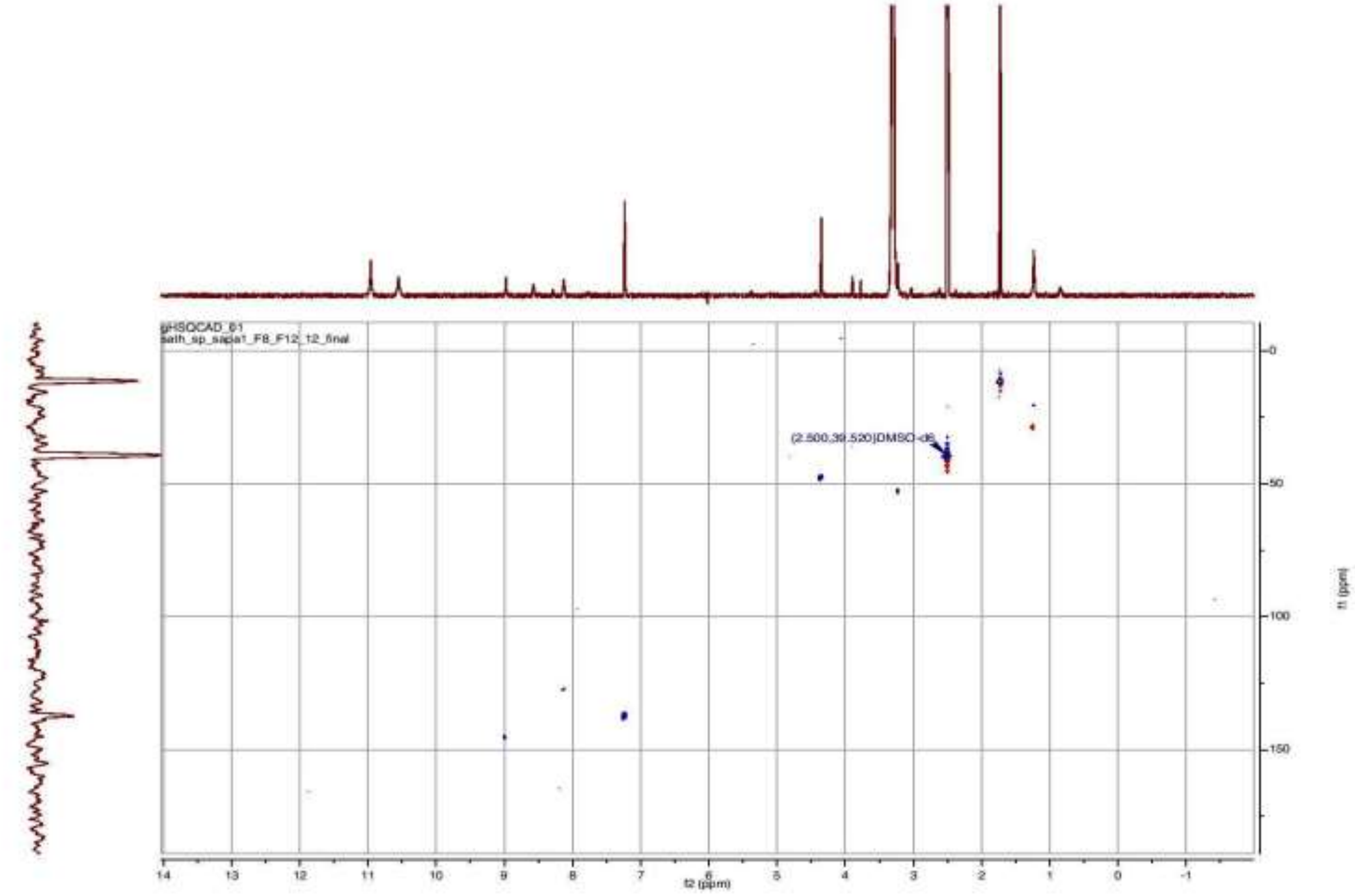

Figure S7c. gHSQCAD of *S. plicata* fraction SP-8 in DMSO- *d*6

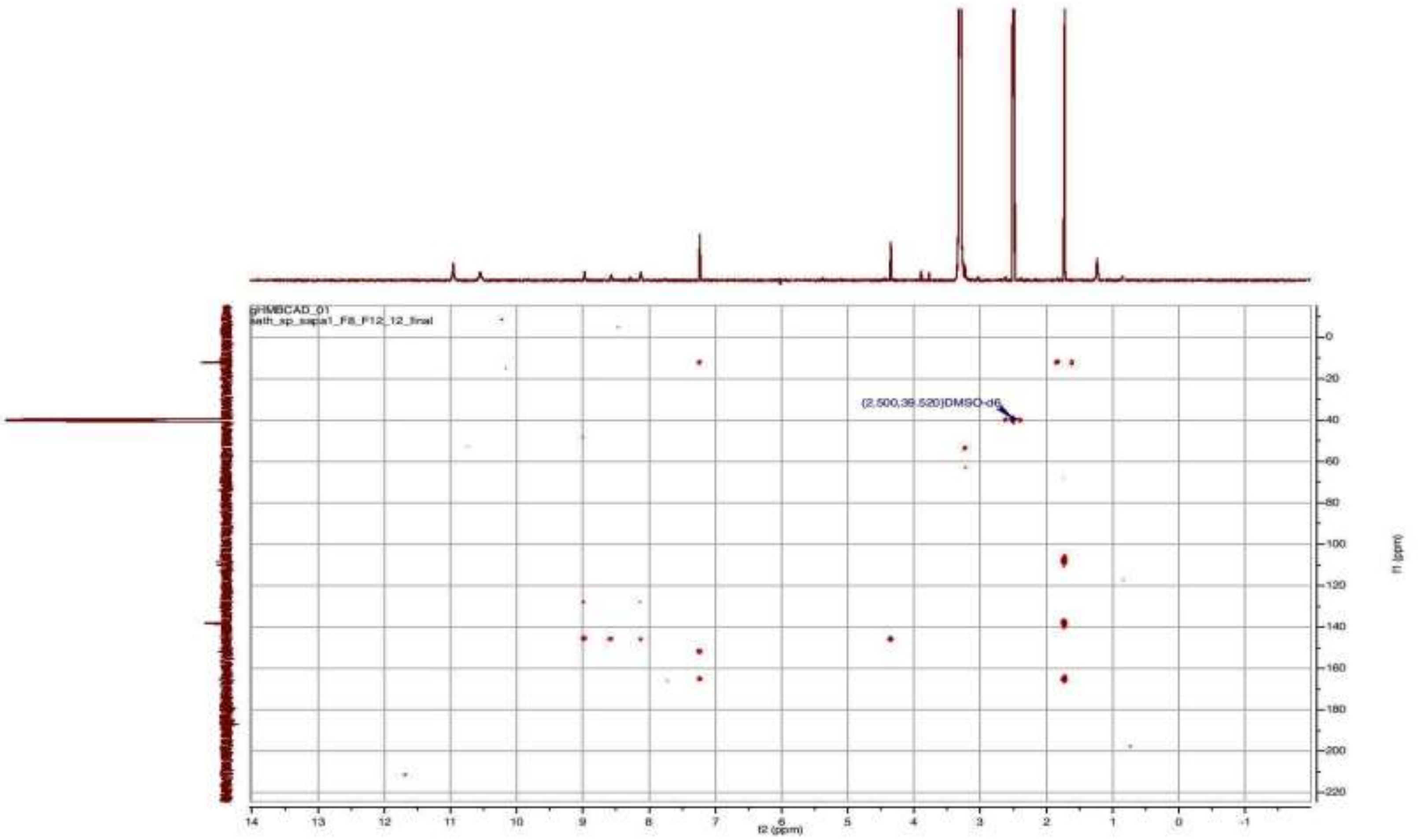

Figure S7d. gHMBCAD of *S. plicata* fraction SP-8 in DMSO- *d*6

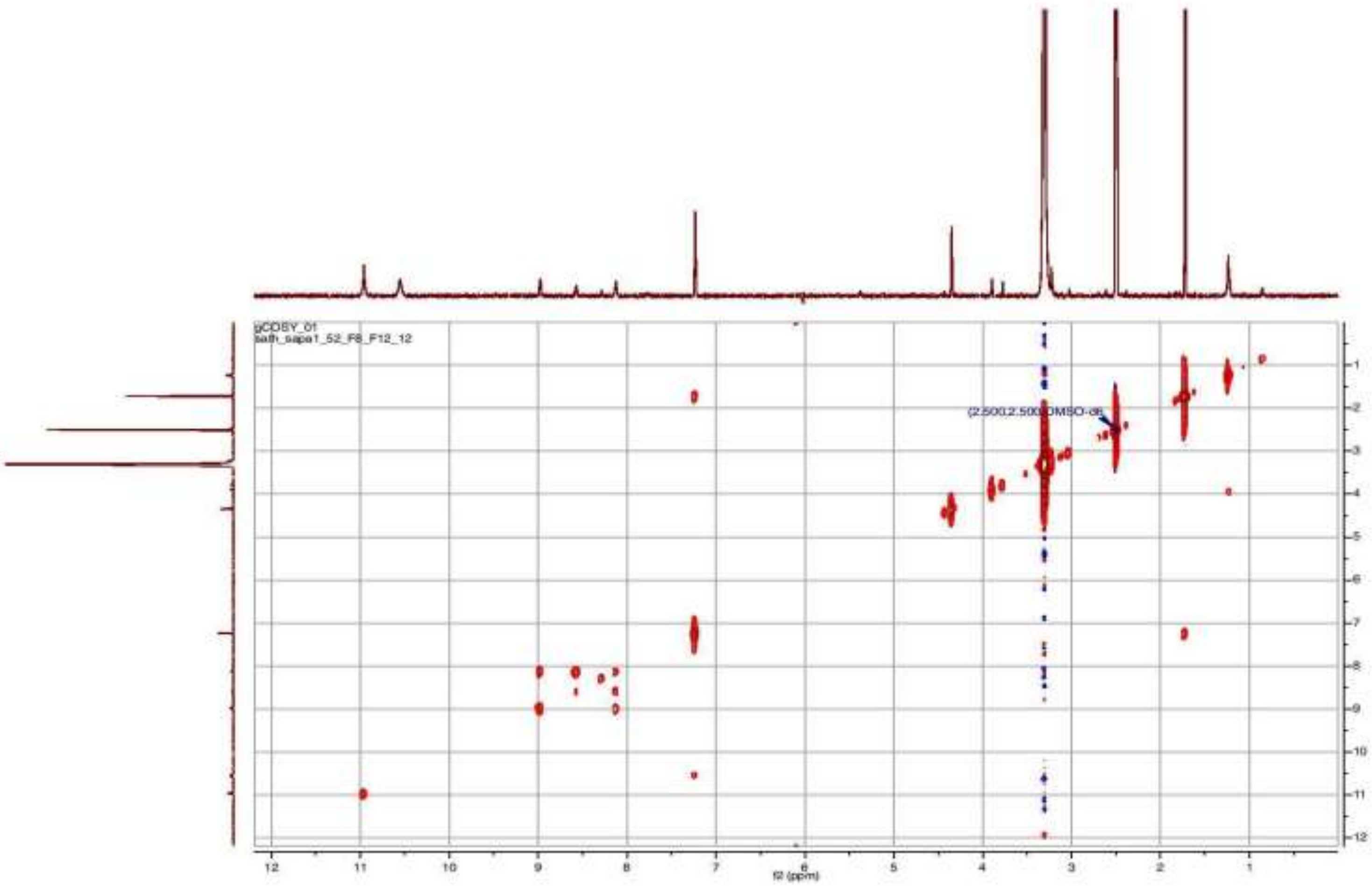

Figure S7e. gCOSY of *S. plicata* fraction SP-8 in DMSO- *d*6

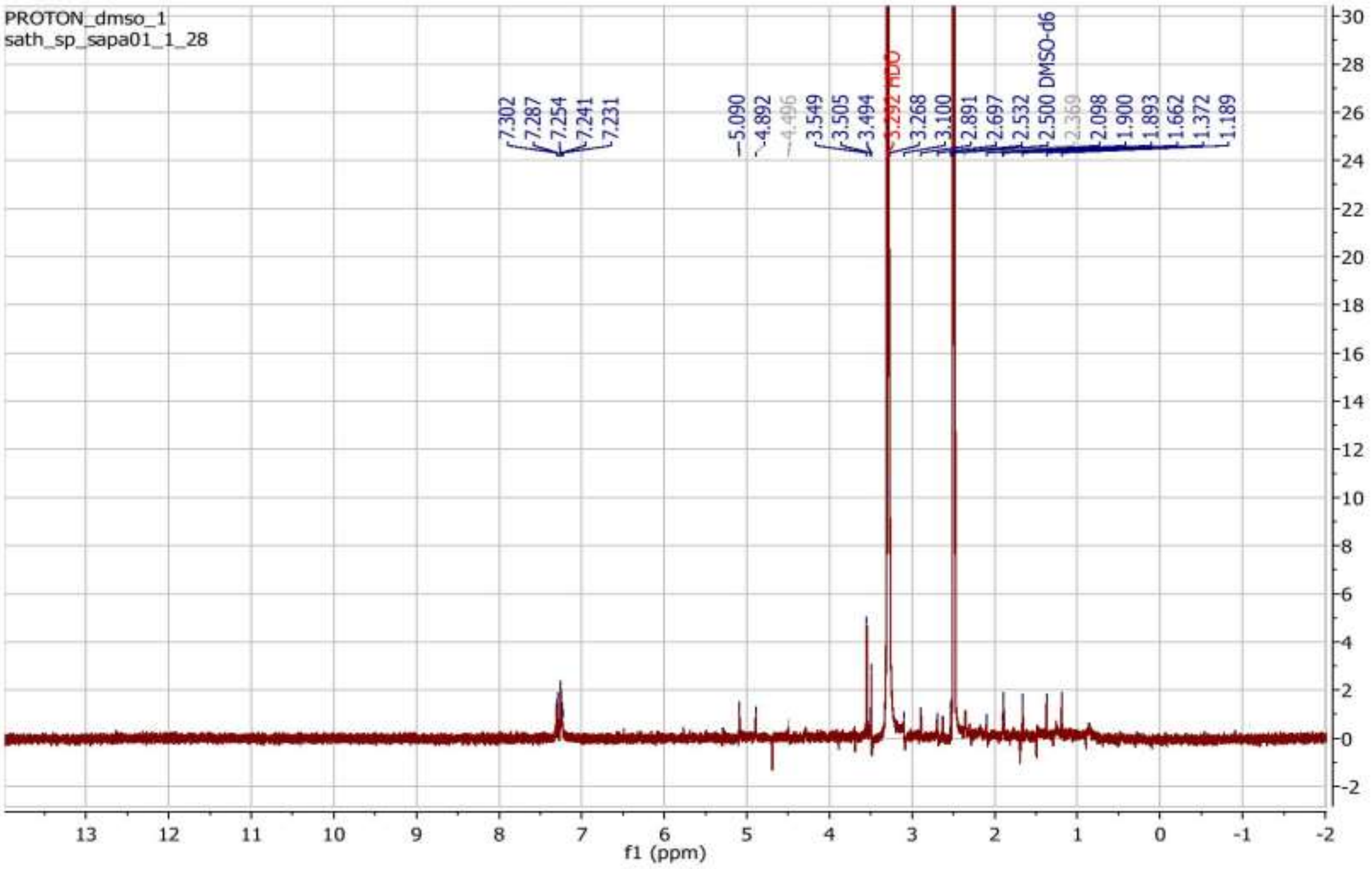

Figure S8. ^{1}H-NMR of *S. plicata* fraction SP-28 in DMSO- *d*6

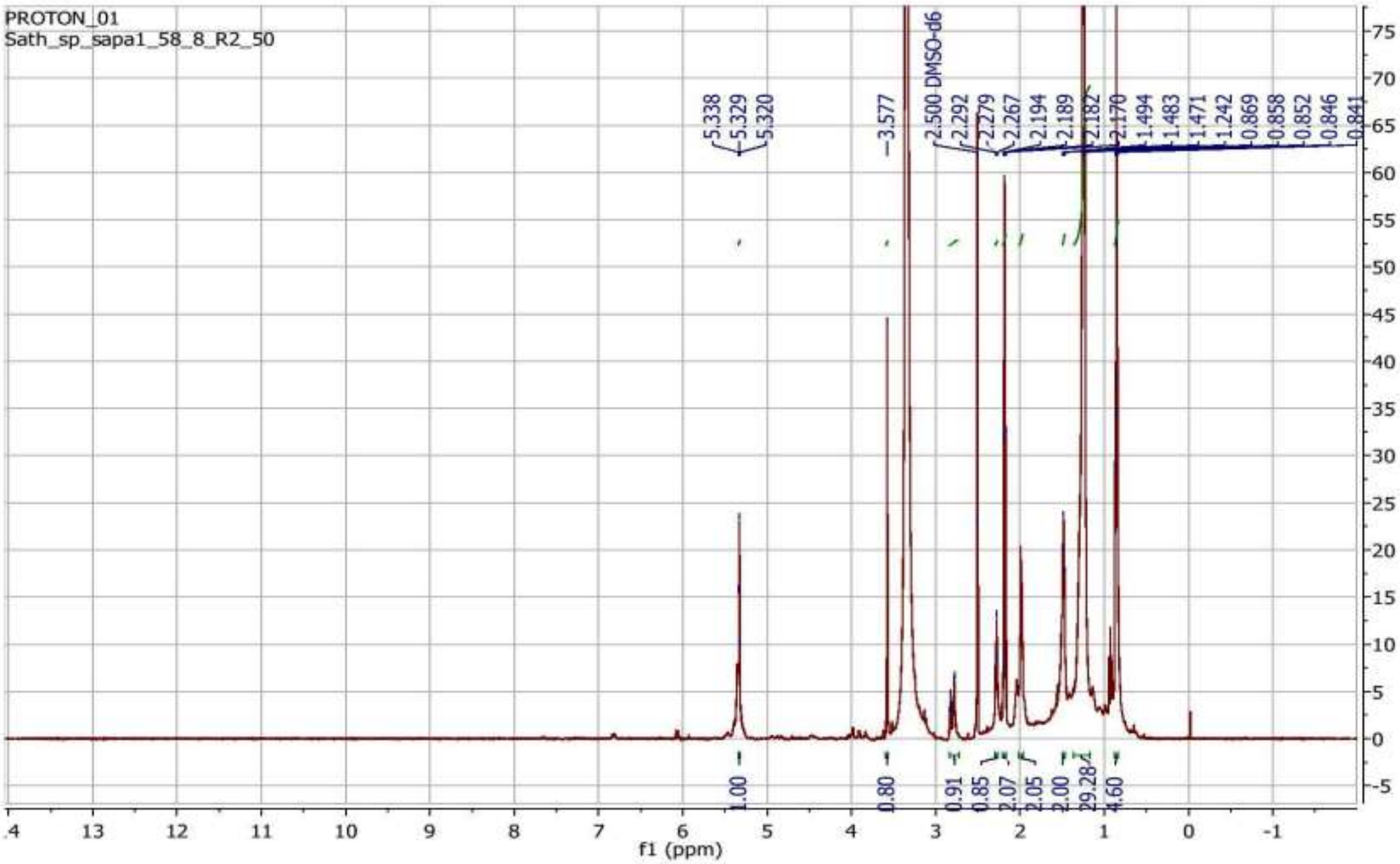

Figure S9a. ^{1}**H-NMR of** ***S. plicata*** **fraction SP-50 in DMSO-** ***d*****6**

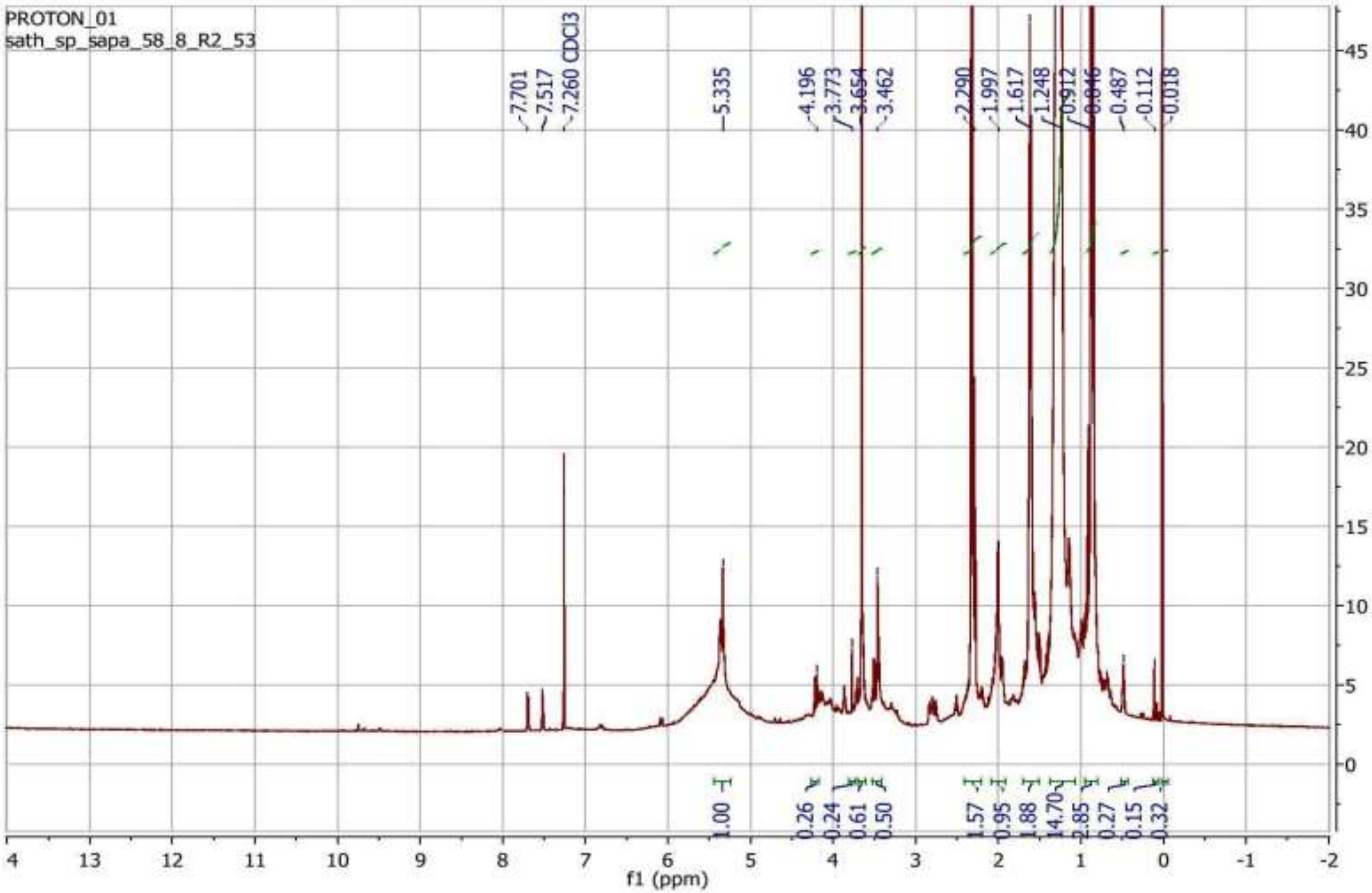

Figure S10a. **^{1}H-NMR** ***S. plicata*** **fraction SP-53 in $CDCl_3$**

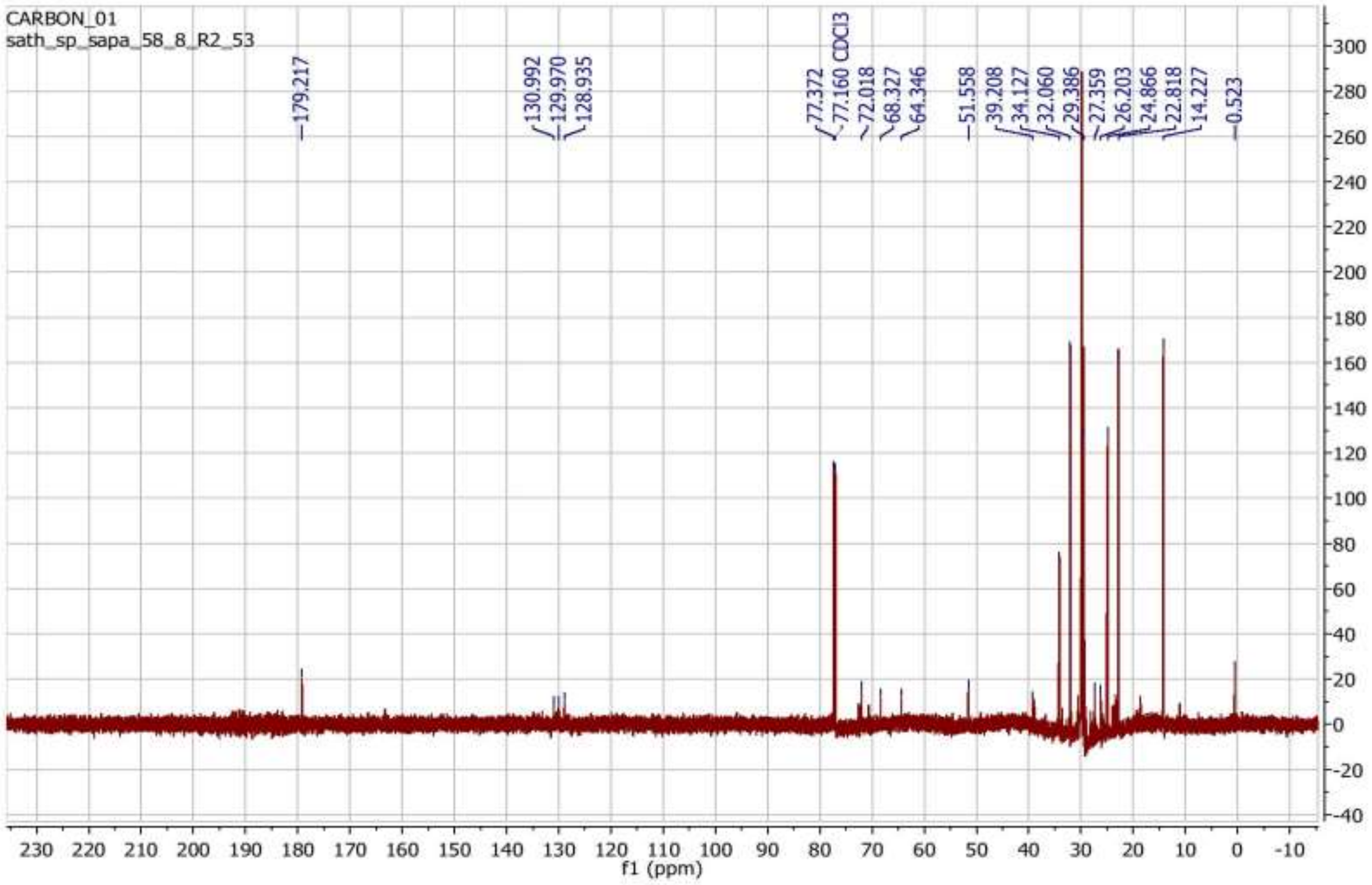

Figure S10b. ^{13}C-NMR of *S. plicata* fraction SP-53 in $CDCl_3$

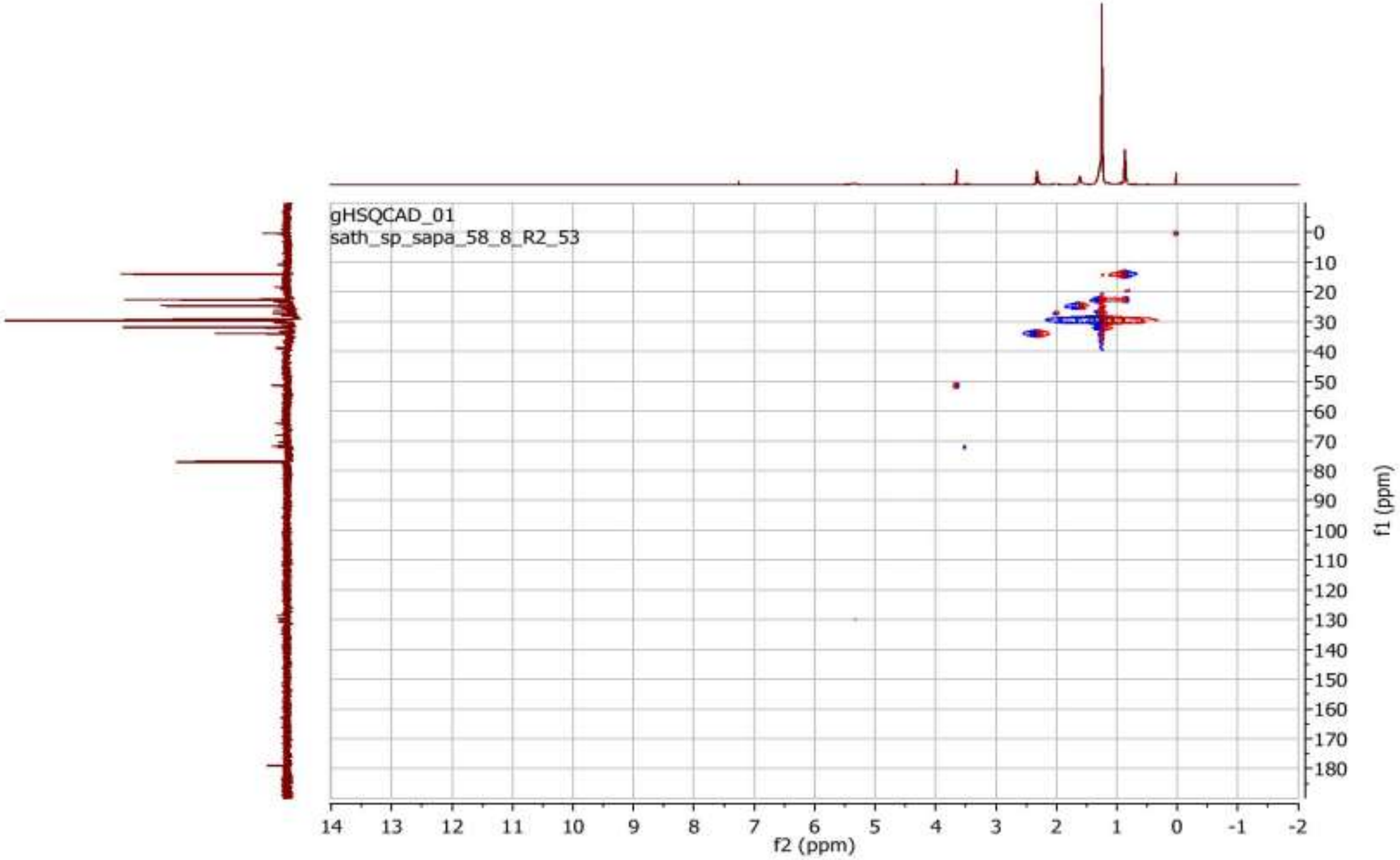

Figure S10c. gHSQCAD of *S. plicata* fraction SP-53 in $CDCl_3$

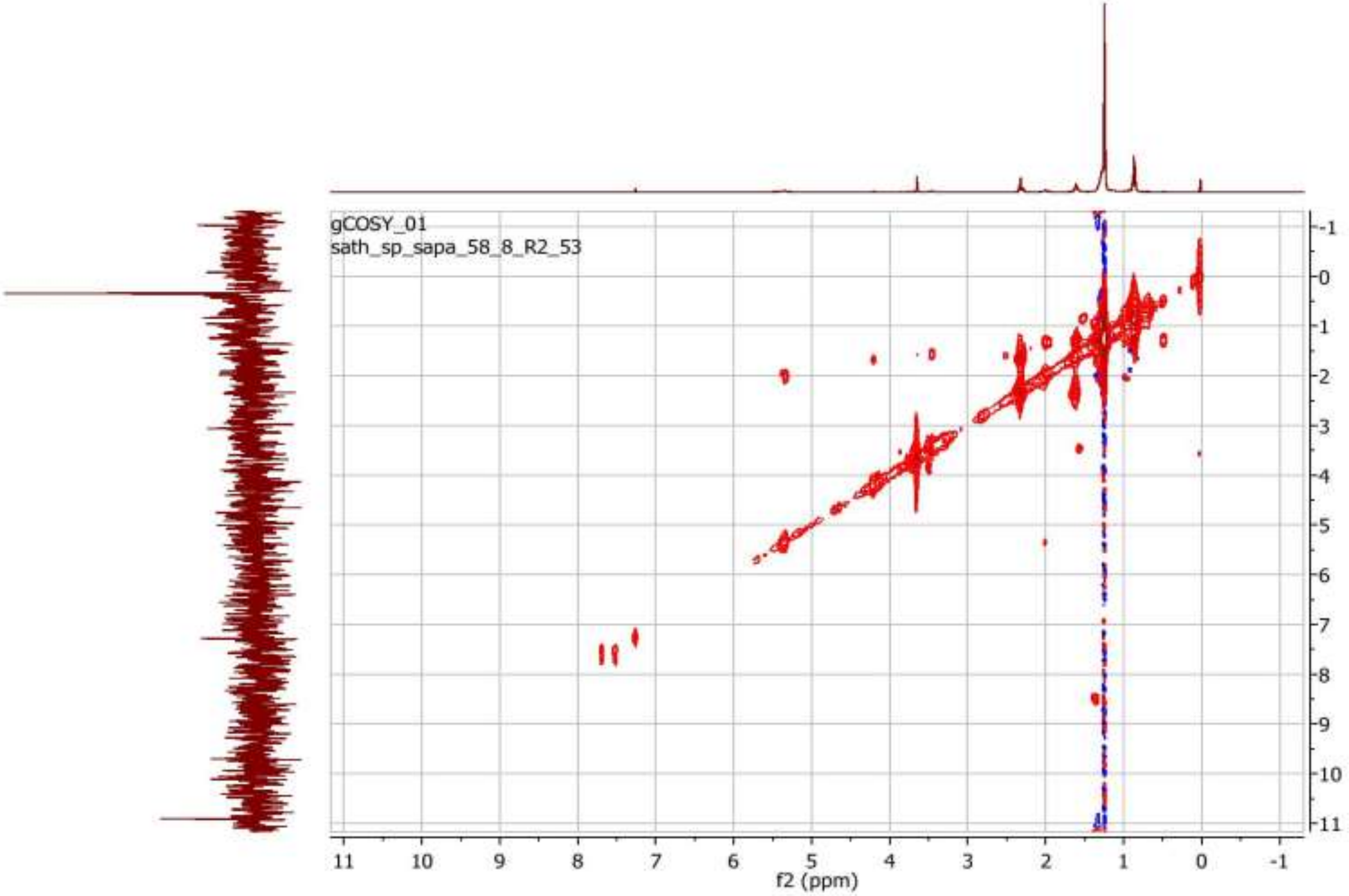

Figure S10d. gCOSY of *S. plicata* fraction SP-53 in $CDCl_3$

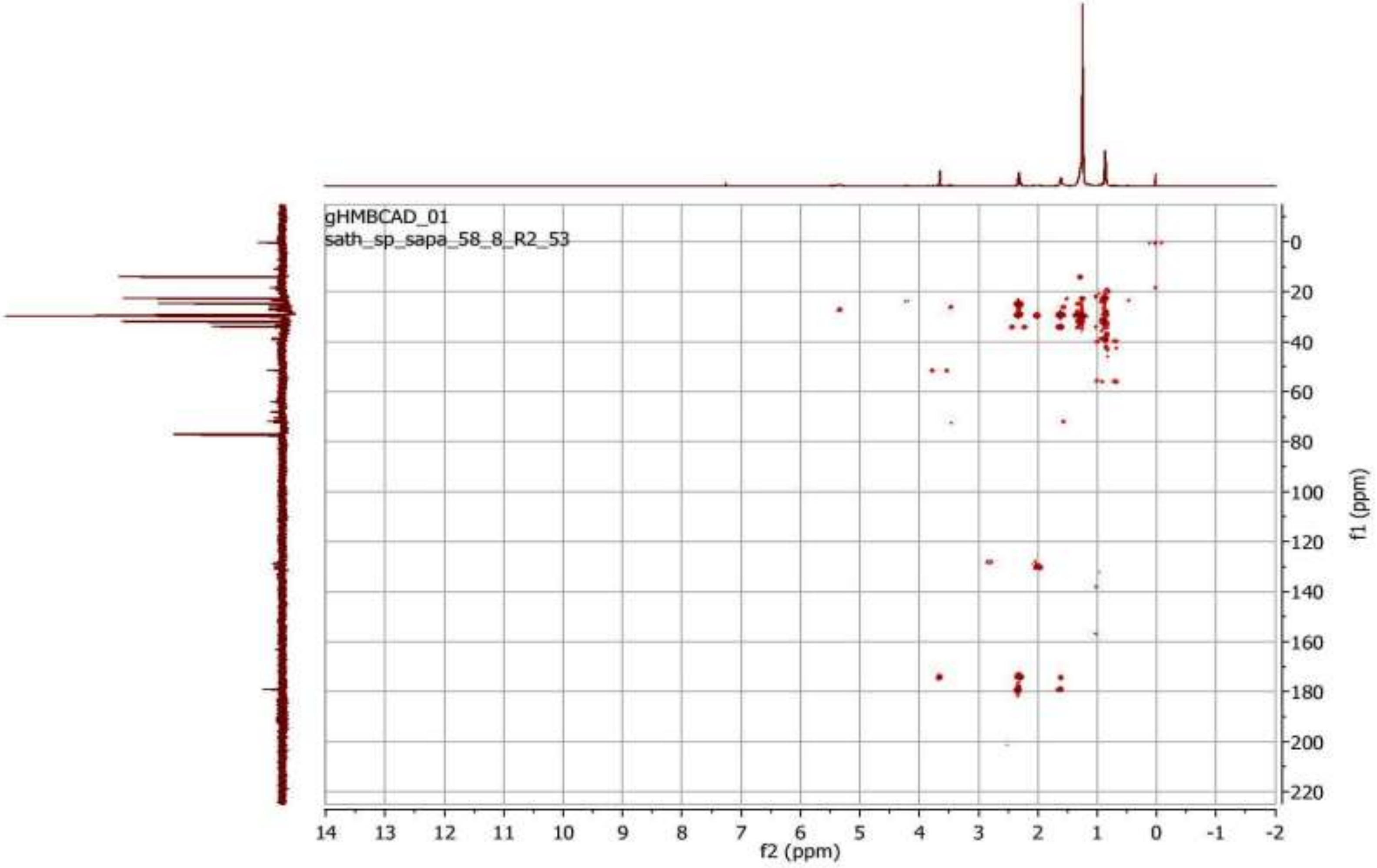

Figure S10e. gHMBCAD of *S. plicata* fraction SP-53 in $CDCl_3$

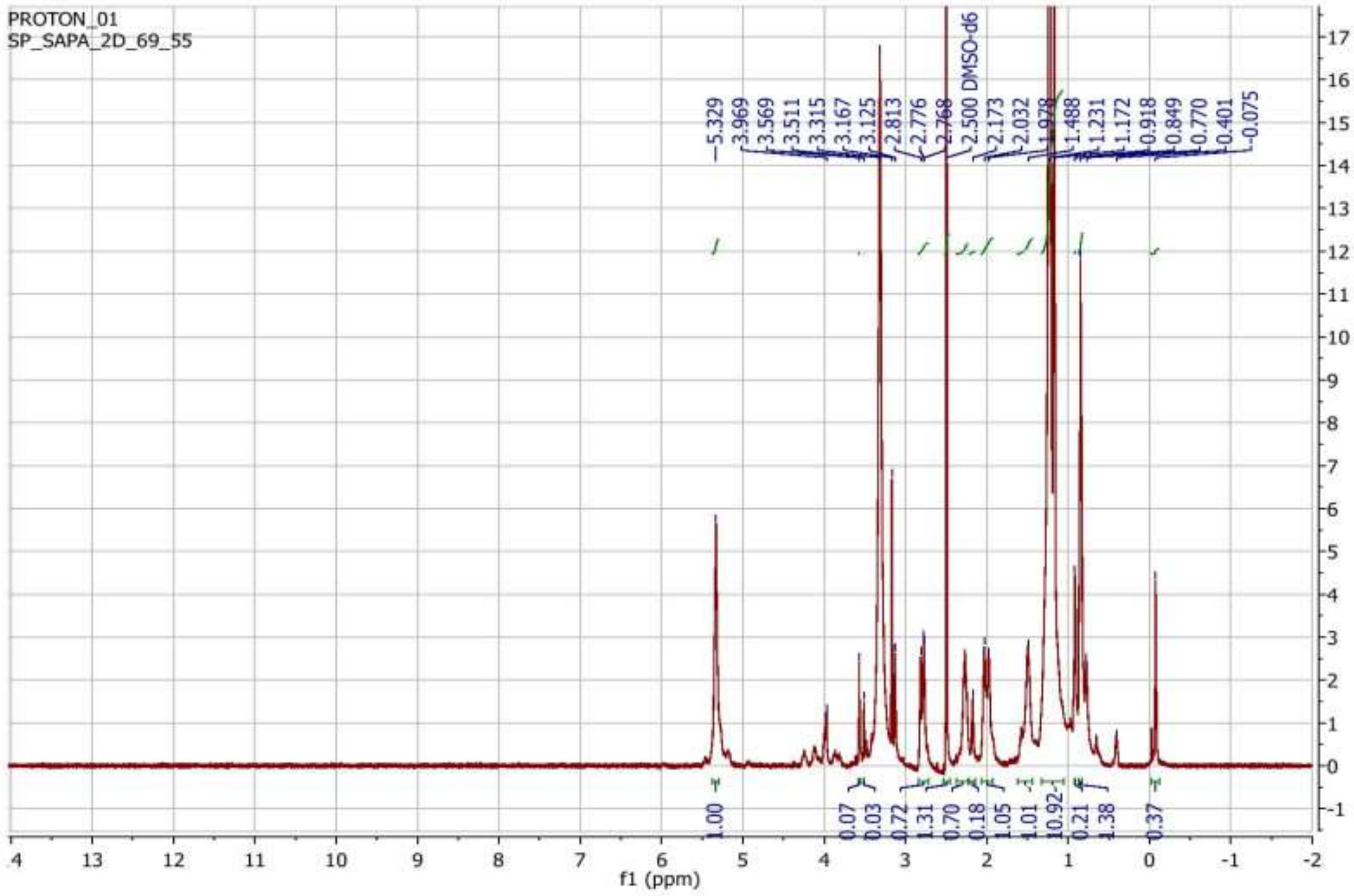

Figure S11. ¹H-NMR of *S. plicata* fraction SP-55 in DMSO- *d*6

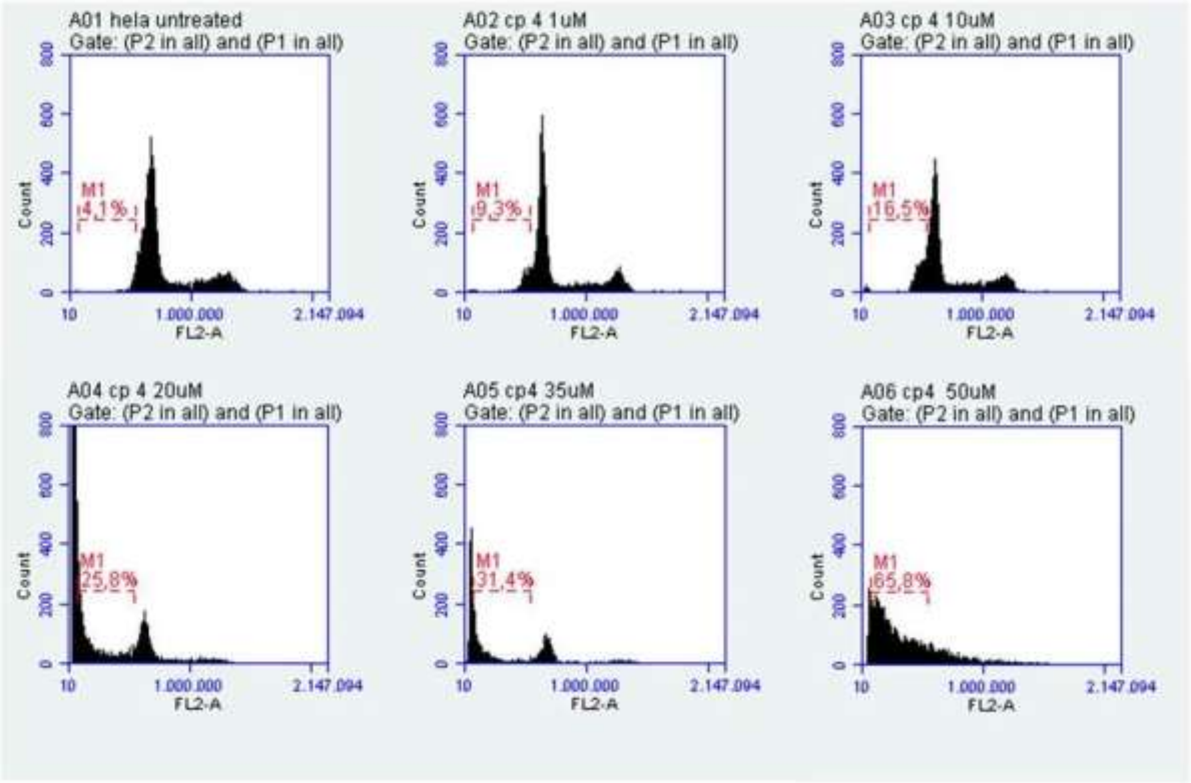

Figure S12. Cell cycle distribution of Hela cells after treatment with fraction SP-50 at concentrations ranging from 1 to 50µM for 72 hrs.

Of note, a dose-dependent reduction of cell viability associated with an increased percentage of cells in Sub-G1 peak observed in both HeLa (Figure S12) and HT29 cells (Figure S13) is indicative of apoptosis. The M1 region of the histogram shows HeLa and HT-29 cells in G_0/G_1 state. Taken together, these data indicate that fraction SP-50 inhibited cell proliferation in both cell lines tested, and that the reduction in the cell viability was associated with induction of apoptosis, being SP-50 the strongest apoptosis inducer with highest growth inhibition against HeLa cells compared to other fractions of *S. plicata*. Based on the analysis, treated cells HeLa and HT-29 Sub-G1 peak showed definite signs of ongoing apoptosis or necrosis.

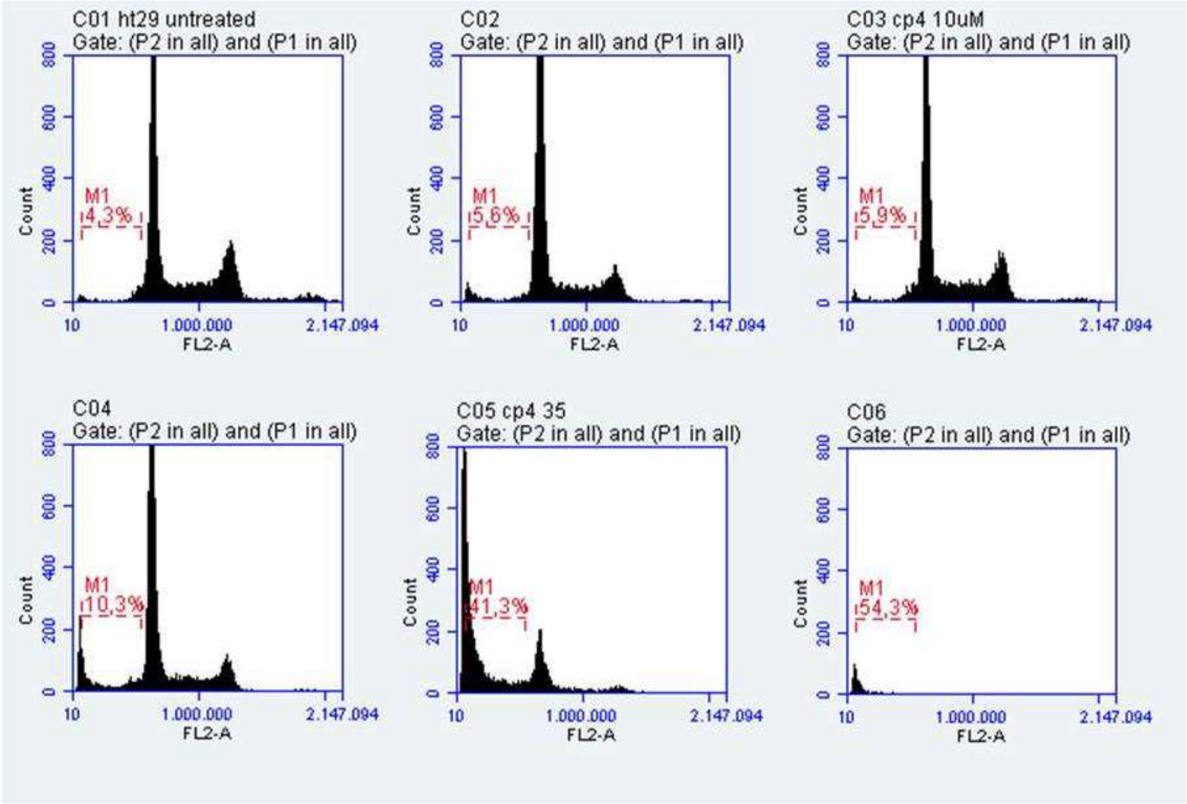

Figure S13. Cell cycle distribution of HT29 cells after treatment with fraction SP-50 at concentrations ranging from 1 to 50µM for 72 hrs.

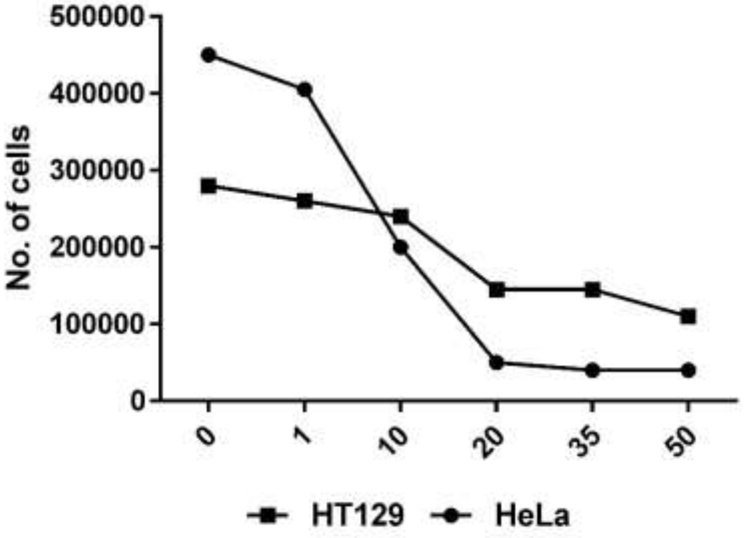

Figure S14. Cell viability of HeLa and HT29 cells after treatment with SP-50 fraction at concentrations ranging from 1 to 50µM for 72 hours
